# AI and Robotics Advancement in Analytical Mineral Characterization and Mining Processes: A Review and Research Trends Analysis

**DOI:** 10.1007/s41061-026-00541-3

**Published:** 2026-03-23

**Authors:** Andile Mkhohlakali, Mothwethwi Priscilla Toona, Tumelo Mogashane, Tshilidzi Rampfumedzi, Portia Madzivha, Mokgehle R. Letsoalo, Napo Ntsasa, James Sehata, Nehemiah Mukwevho, Thembakazi Ncedo, Mothepane Happy Mabowa, James Tshilongo

**Affiliations:** 1 Analytical Chemistry Division, Mintek, Randburg, 2194 South Africa; 2 School of Chemistry, University of the Witwatersrand, Johannesburg, 20250 South Africa

**Keywords:** AI machine learning tools, GIS remote sensing, Bibliometric analysis, Mining processes, Analytical mineral characterization

## Abstract

The mining sector is undergoing a major transformation, as it moves shifting from traditional, labor-intensive methods to adopting digital technologies within the framework of Industry 4.0. Machine learning (ML), artificial intelligence (AI), and robotics are emerging as key innovative tools to improve safety, operational efficiency, and sustainability across the entire mining value-chain, from exploration and mineral processing to mineral characterization and environmental management. The integration of AI and ML with spectroscopic techniques has revolutionized the mining industry by enhancing efficiency, accuracy, throughput, and operational performance. This review discusses recent advances in AI, ML, and robotics applications in mining processes and mineral characterization. It explores the influence and highlights the integration of ML tools such as ANN, PCA, k-NN, and SVM with advanced analytical chemistry techniques, including XRF, XRD, SEM–EDX, LIBS, ICP-OES, ICP-MS, LA-ICP-MS, and HSI for mineral identification. Additionally, a bibliometric analysis using Scopus publications over the past 20 years provides insights into research trends and hotspots, providing recent insights into publication patterns and research. The review further offers an overview of recent technological developments, economic benefits, policy implication changes, and future directions, while emphasizing gaps related to the standardization of prospects for mining, demonstrating substantial growth in the integration of AI-driven analytical technologies within the analytical chemistry characterization of minerals, while also highlighting gaps related to the standardization of technologies.

## Introduction

**Fig. 1:**
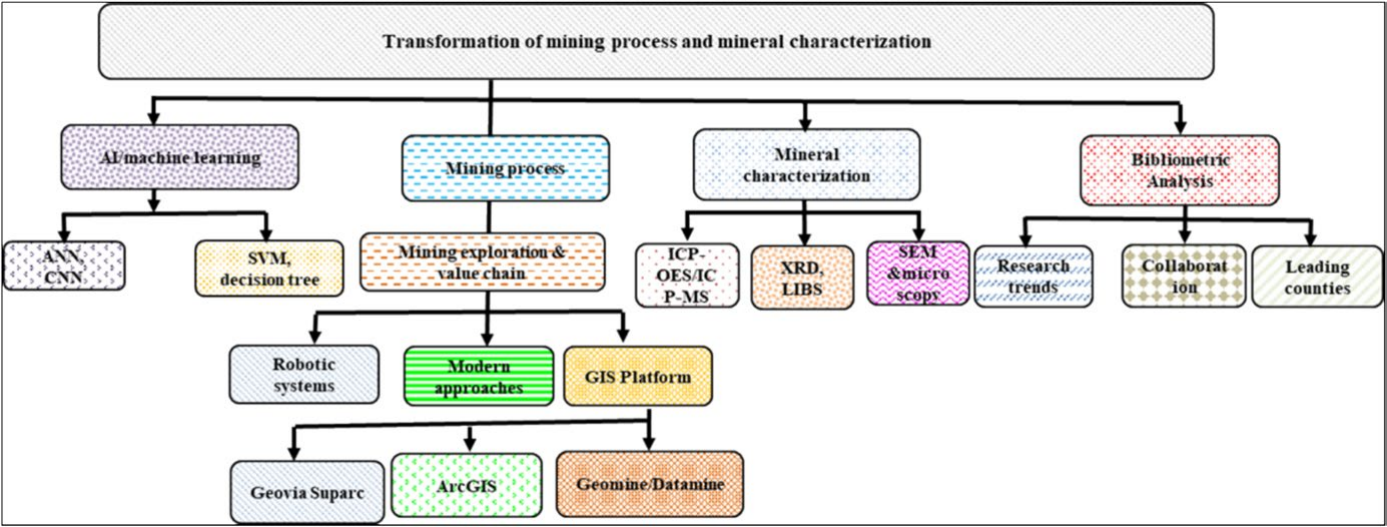
Flowchart of the content, emphasizing key topics discussed

The global mining sector is rapidly transitioning from manual, labor-intensive processes (i.e., during of the 2nd Industrial Revolution) to the use of the digital technologies of the 4th Industrial Revolution (Mining 4.0) [1]. This transition shift is driven by the increasing operational complexity of mining operations, the rising demands for increased production, and the requirement to comply with stricter environmental regulations [2, 3]. The new approach of digital technologies enables real-time monitoring, intelligent systems, and automation control to mitigate hazards and reduce labor-intensive tasks [2]. Generally, the mining process and mining value chain generally includes (1) exploration, i.e., finding the mineral (ore discovery), (2) drilling and blasting, i.e., separating the ore extraction from the rock, (3) crushing, milling/comminution, and (4) separation, extraction, and refining [2, 4]. The integration of artificial intelligence (AI) and machine learning (ML) in the mining sector is now being utilized, from ore body modeling and exploration and modeling the ore body prediction, to mineral processing (crushing, comminution), mineral separation, environmental monitoring, and end-of-life activities, such as tailings management [5]. By analyzing large geological datasets, AI and ML algorithms are being used to analyze the big geological data and enhance the accuracy of the ore body prediction. These techniques utilize the decision tree (DT), support vector machines (SVM), artificial neural networks (ANN), principal component analysis (PCA), and *k*-nearest-neighbor (KNN) models to execute the modeling of complex geological data or systems. In addition, these technological approaches offer significant potential to reduce mining operation risks, optimize resource use, and enhance product quality capabilities [6]. Consequently, in parallel, robotics are transforming the mining sector through the deployment of automated vehicles, robotic drilling systems, and plant-based automated samplers, These technological tools focus on improving operational accuracy and precision while also reducing the safety hazards in both open-pit and underground mines. Figure 1 summarizes the key topics covered and discussed in this review, including for advancements of mining process transformation and mineral processing, complemented by bibliometric trend visualization monitoring.

**Fig. 2:**
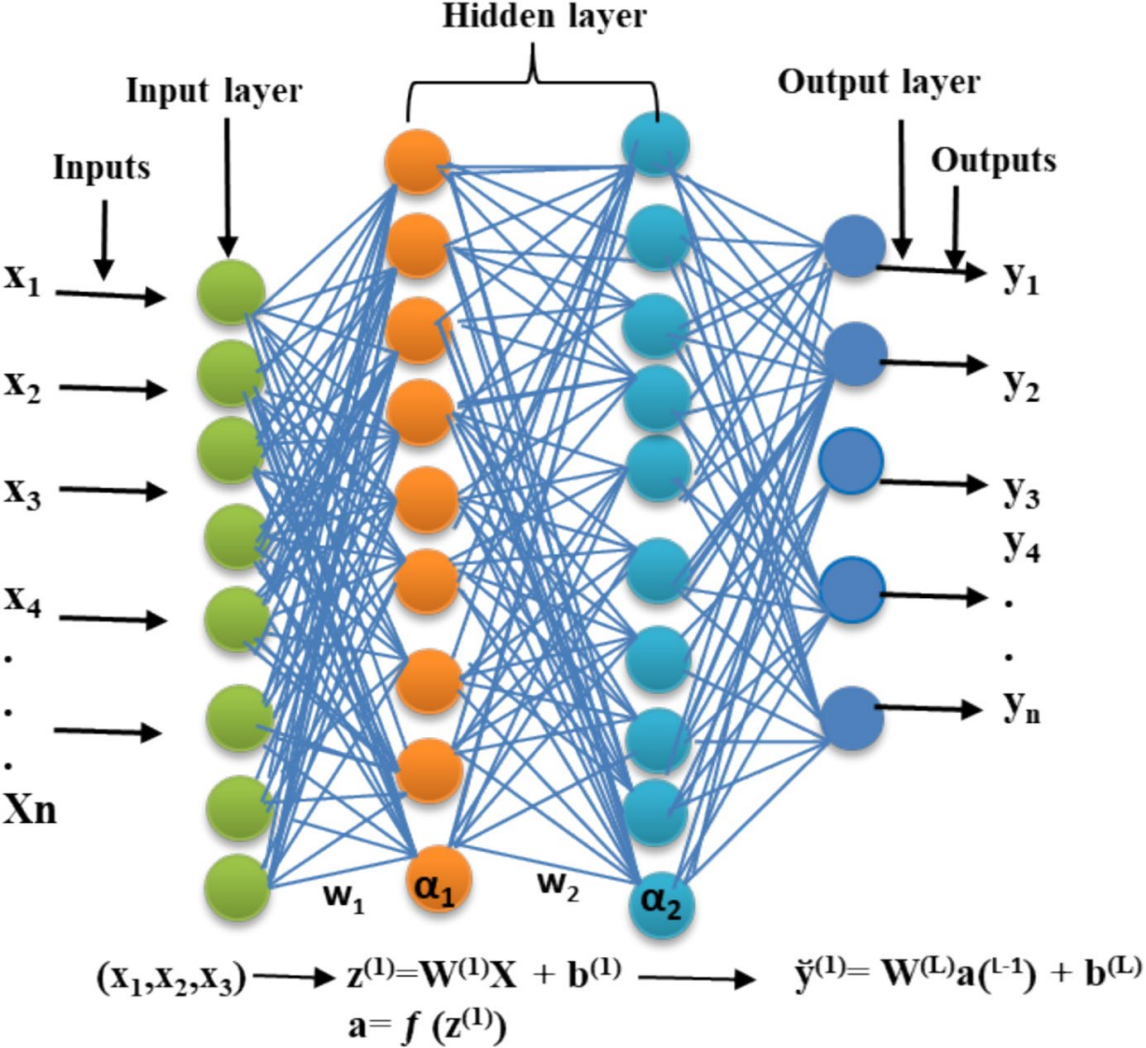
Schematic of a layered feed-forward ANN and its working principle

The integration of AI and ML tools in analytical chemistry has been widely applied, especially in spectroscopy, particularly in techniques in various fields such as the food industry, science, and chromatography [7]. However, the use of ML logarithms in mineral characterization has received comparatively less attention. The application of AI/ML in the analytical characterization of minerals has revolutionized the mining industry by improving precision in data analysis and monitoring of mineral processing. In mineral characterization, advanced instrumentation techniques such as laser-induced breakdown spectroscopy (LIBS), X-ray fluorescence (XRF), inductively coupled–plasma optical emission spectrometry (ICP-OES), inductively coupled plasma–mass spectrometry (ICP-MS), laser ablation–inductively coupled plasma–mass spectrometry (LA-ICP-MS), X-ray diffraction (XRD), scanning electron microscopy combined with energy dispersive X-ray spectroscopy (SEM–EDX), and hyperspectral imaging (HSI) generate complex, large, datasets [8, 9]. ML and deep learning (DL) employ algorithms to enable faster analysis and to automate data interpretation, enabling large datasets, facilitating mineral classification, quantification, association, and structural analysis [10]. Additionally, DL further enhances peak identification, spectral quality, and noise reduction in various spectroscopic applications [11]. These capabilities facilitate the use of sophisticated tools allowing for mineral classification and anomaly detection with minimal human intervention. Consequently, the integration of advanced instrumentation with AI is giving rise to 'smart labs', which are emerging through the integration of advanced analytical techniques and instrumentation, resulting in faster and more precise mineral assessments. These technologies support real-time decision-making, thereby improving exploration efficiency and ultimately transforming traditional methods of mineral analysis [12]. However, challenges remain in applying AI and robotics to mining and mineral characterization, including high capital costs and the need for specialized expertise. AI methods and robotics often utilize artificial neural networks (ANNs), which are widely used in DL applications. Among the most common tools arer ML algorithms used to solve DL problems. As shown in Fig. 2, ANNs operate in a feed-forward manner using multilayer perceptions (MLPs), which consist of one or more hidden layers of neurons [13]. The neurons (*α*) receive raw input data (× 1, × 2, …, *xn*)^T^ and process them through activation functions capable of capturing non-linearity [14]. To minimize errors, the training process involves back-propagation, (feedback) to adjusting the weights (*W*) and bias (*b*) to minimize errors associated with each neuron connection. In visual terms, a feed-forward ANN can be expressed as input (× 1, × 2, × 3….*xn*)^T^, a hidden layer: *z*(1) = *W*(1)*x* + *b*(1), *a*(1) = *f*(*z*(1)), and an output layer, *y* = *W*(*L* − 1) *a*(*L* − 1) + *b*(*L*), where *x* denotes the input parameters, *W*(*l*) and *b*(*l*) are weights and biases, for layer *l,* ƒ(.) represents activation functions such as, e.g., sigmoid, and ReLU, and y represents the final output. Analytical chemistry generates large chemical datasets and large complex datasets that AI, particularly MLPs, can process efficiently for data management, interpretation, and mineral characterization [8].

Thus, in this review, we will discuss recent advances in geographic information systems for mineral exploration, and the integration of spectroscopic techniques with ML and AI for applications in analytical characterization of mineral characterization and mining processes. Furthermore, discussion of it also presents a bibliometric analysis to identify the research hotspots and major patterns in research trends in keyword usage across publications over the last 20 years, from 2005 to 2025.

Table 1 summarizes the review articles that focus on trends in AI application in analytical chemistry, trends in particularly drug analysis, the food industry, and AI application in general analytical chemistry. The main gap lies in the lack in AI applications in the analytical chemistry of mineral characterization and the mining value chain (mining from exploration to analytical characterization). Although AI indicates a positive growth in extractive/geometallurgy, mineral ore processing, and mineralogy, discussion of AI applications in analytical mineral characterization remains unexplored. Additionally, existing reviews address different analytical techniques like ICP-OES, ICP-MS, XRF, XRD, LIBS, and HSI in mineral ore characterization, but none provide a consolidated side-by-side evaluation of AI integration, and there is no consolidating review article reporting such an evaluation of AI and spectroscopic techniques for mineral characterization. This paper is unique in also exhibiting the uniqueness for consolidating AL/ML tools in analytical techniques for simplifying the large, complex datasets, improve accuracy, enhance efficiency, and increase throughput. It also introduces AI application tools in the mining value chain (from exploration to mineral analysis). The technological trends are viewed and discussed through bibliometric analysis, integrating using VOS Viewer and R-studio, analyzing the trends over the past 20 years. To the best of the authors’ knowledge, this integrating of technical discussion with bibliometric visualization, for the first time provides a narrative snapshot of current trends/landscape and future directions of mining processes.

**Table 1:** Summary of side-by-side comparison of AI integration with analytical chemistry applications

Research activities and gaps	References
General application of AI tools in analytical chemistry. However, there is no specific focus on mineral characterization or the mineral value chain and there is a lack of AI integration with spectroscopic techniques across the mineral value chain	[8]
Deep learning approaches in analytical chemistry. Nonetheless, there is less focus, and is limited to mineral characterization and no mining applications	[9]
Highlights the integration of AI/ML with spectroscopic application in coal mining. There is no broad application of AI integration in mineral characterization	[10]
Significant impact of AI integration with spectroscopic application in for food quality and safety monitoring. The study is limited to food quality-related studies without non-mineral application-related utilization	[7]
Demonstrates the incorporation AI in robotics for chemical synthesis and reaction discovery. The research is narrowly focused on organic synthesis with no focus on mineral relevance analysis	[11]
Highlights the utilization of deep convolutional neural networks with LIBS, addressing the technical challenges in achieving accuracy for multicomponent quantitative analysis	[12]
Consolidation of AL/ML tools in analytical techniques for simplifying the management of large, complex datasets, improving accuracy, efficiency. and throughput. Also introduces AI application tools in the mining value chain (from exploration to mineral analysis). The technological trends are reviewed and discussed using bibliometric analysis integrated with VOSviewer and R-studio, analyzing trends	Our work

## Bibliometric Analysis of Mineral Processing and Characterization

Bibliometric analysis is a quantitative analytical tool used in the field to detect and analyze the numerous research trends that have been undertaken in several disciplines, including science and engineering [13]. It allows researchers to access tools enabling academics to evaluate publishing patterns, trends, citation metrics, and collaboration networks, offering insights into the evolution and future directions of specific subjects [14]. While the method of bibliometric analysis is not new, its use and application has expanded across many sectors in recent years. Because it uses quantitative approaches provides data about article authorship, collaborations, publishing countries, citations, and several bibliometric networks, while also mitigating interpretation bias [13–16]. For bibliometric analysis and visualization, researchers use tools such as VOSviewer, the R-package *bibliometrix* and its web interface (i.e., Biblioshiny) [16]. Biblioshiny enables researchers to use its integrative functions to conduct bibliometric analysis and scientometric analyses from imported datasets, converting them into diverse different analytics and visualizations across multiple plots for various matrix levels, offering insights into the evolution and future orientations of specific subjects [14]. The descriptive statistical analysis from Biblioshiny includes citation counts by author, affiliation, and state (countries).

**Fig. 3:**
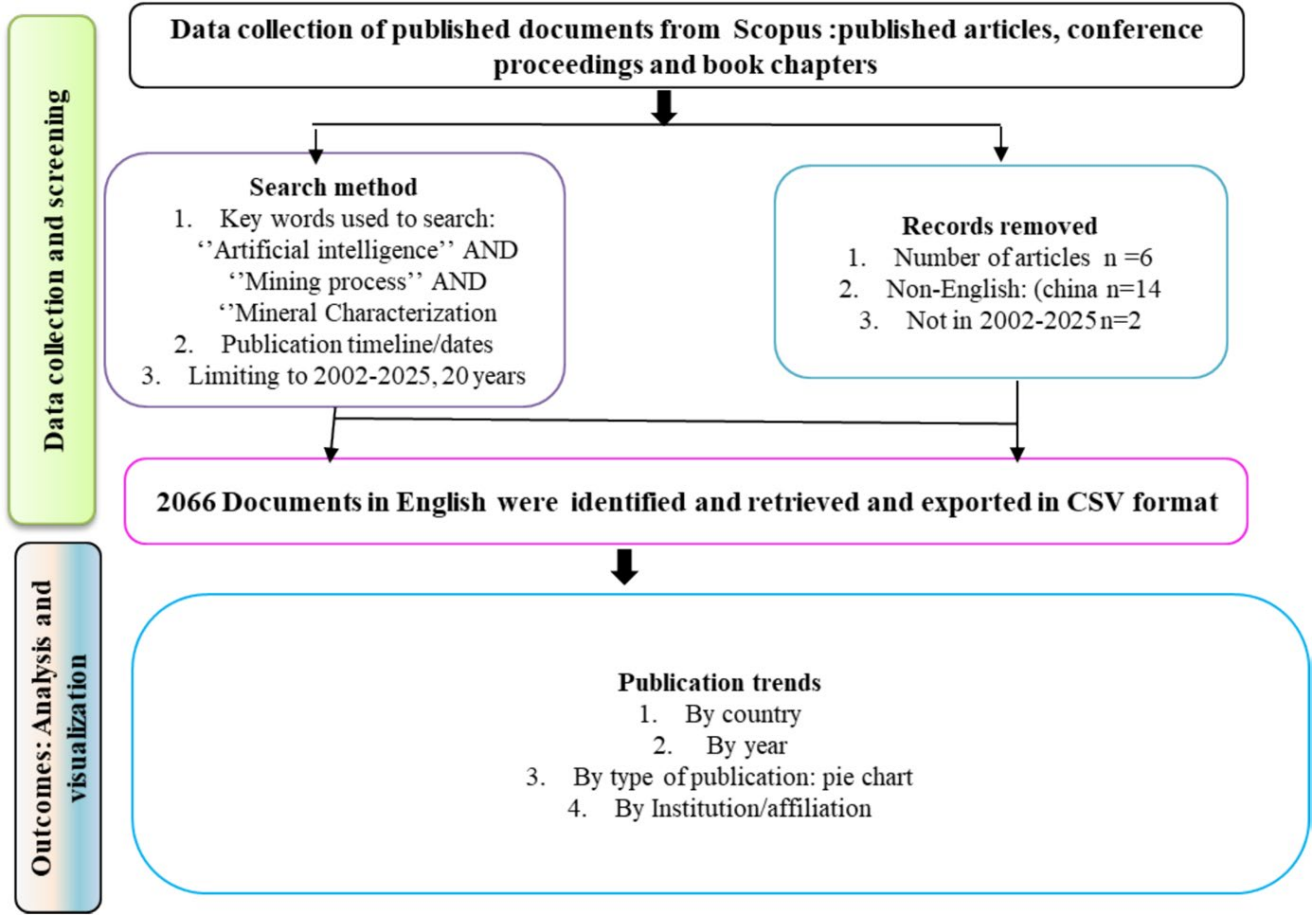
Flow diagram of AI in mining process and characterization literature and screening

For this study, the literature data were retrieved from the Scopus database, including research articles, conference papers, book chapters, and books. Moreover, keywords were applied to search articles for a bibliometric analysis, “Machine learning”, “Mining processes”, and “Analytical mineral characterization”, which identified key research trends in areas such as ML, reservoir characterization, ANNs, seismic processing, drilling, geothermal processing, digital mapping, and health risk, as illustrated in the word cloud (frequency analysis search). The aim of this review was to assess the research hotspots, international collaborations, and key players (leading countries) in AI and robotics in mineral processing and mineral characterization. The research dataset was retrieved and exported from the Scopus database on 10 May 2025,. As revealed in Fig. 3, previous studies' datasets show the timeframe from 2005 to 2025 (20 years). A total of 2062 research-based publication outputs on the above-mentioned subjects were retrieved.

### Methodology

#### Data Collection and Screening

The data collection and screening was done using SCOPUS from the Scopus database involving four phases: (1) identification of relevant published documents (i.e., journal articles, conference proceedings, books, and book chapters) related to AI in mining processes and mineral characterization; (2) restriction to English-language publications; (3) export of data using CSV format for loading in computer program softwares such as VOSviewer and Biblioshiny (R-Studio), and (4) analysis to assess the documents by year, country, and affiliation. The flow diagram in Fig. 3 summarizes the process of data collection and screening by publication outputs, leading nations, document types, annual production, and researchers’ affiliations.

#### Bibliometric Analysis Preliminary Data from SCOPUS

**Fig. 4:**
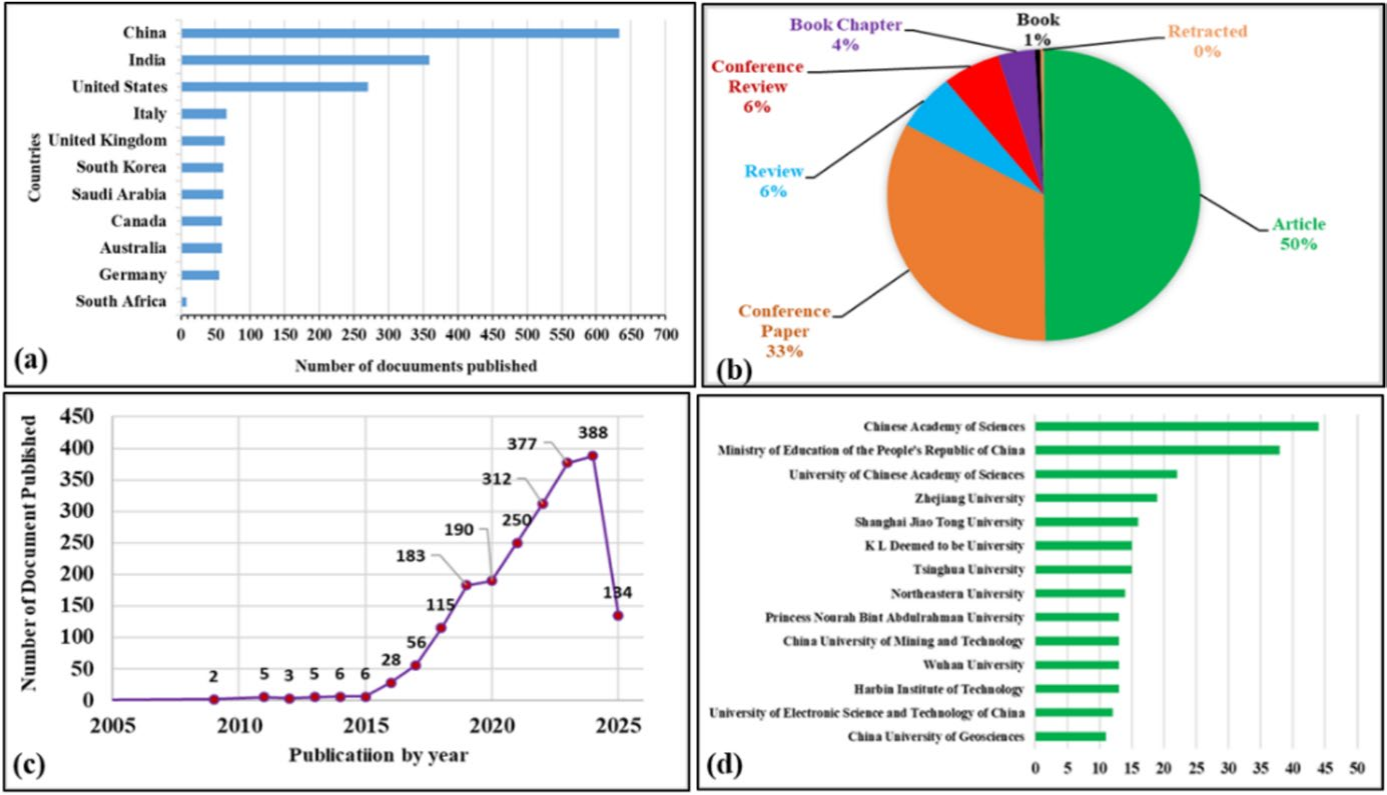
Bibliometric analysis of the published documents on AI and robotic in mining process and mineral characterization, indicating **a** publication outputs by year, from 1995 to 2025, **b** document types, exhibiting the proportion of journal articles, conference proceedings, journal reviews, book chapters, books, and retracted papers

As shown in Fig. 4a, publications from 2005 and 2025 by country indicates that the outputs are dominated by countries with advanced technology and by mining-intensive countries. The leading country in publication by volume is China, with approximately ~ 650 outputs, followed by India and the United States [17]. Strong research funding, academic institutions, well-established industry collaboration, and robust industry infrastructure could contribute to these trends. Notably, despite South Africa being a developing country rich in mineral wealth, its contribution remains limited, providing little research capacity and with restricted access to AI and robotics related to mineral processing and characterization, thus mineral richness does not translate into research leadership and activities. The trends of developing countries suggest that entry barriers are increasing as the research area matures, moving towards capacity building and targeted investment more crucial to avoid technological dependency on developed countries. Additionally, this implies a potential risk of widening the technological asymmetry where developing countries like South Africa increasingly adopt AI tools rather than refocusing research in context-specific solutions. These data suggest the world’s concentration of expertise, which underscores the importance of international collaboration [18]. Mapping the distribution of publications highlights research trends, hotspots, and gaps where enhanced academic and policy support could promote balanced development in the field of AI in robotic mining processes and mineral characterization. Figure 4b indicates that most of the published documents are journal articles, followed by conference papers and reviews. The development of books and book chapters continues, making up 4% and 1% of the total publications, respectively [19]. The latter results present the opportunity for developing countries to contribute and showcase their research activities through applied research, peer-reviewed journals, conference proceedings, and book chapters in region-specific challenges. Typically, the percentage shown in the pie chart is lower at 33%, but it still provides critical synthesis and guidance for future research paths. The overall combination of these documents suggests both technological innovation and academic (scholarly) maturity. Figure 4c) displays published documents from 2005 to 2025, revealing the sharp increase in research publications, especially post-2015, which is ascribed to advancements in AI technologies and increased interest from the industry in the digital transformation of the mining industry and mineral characterization. The steady increase post-2020 is due to automation needs during the COVID-19 pandemic [20]. The publication of approximately 380 documents between 2021 and 2024 indicates a growing academic and industrial recognition of robotics and AI in mining as transformative tools. The contributory affiliations in Fig. 4d show a small number of institutions that are leading research on AI and robotics in mining, processing, and mineral characterization. The top contributors include the Chinese Academy of Sciences (44), followed by the Ministry of Education of the People’s Republic of China (36) and the University of Chinese Academy [21]. The limited representation of institutions from developing countries highlights opportunities for broader research participation aimed at capacity building. To better illustrate the evolution of research activity in this field, Fig. 4 presents the increasing number of publications related to AI and robotics in mineral processing between 2010 and 2024.

#### Extraction of Data and Analysis

**Fig. 5:**
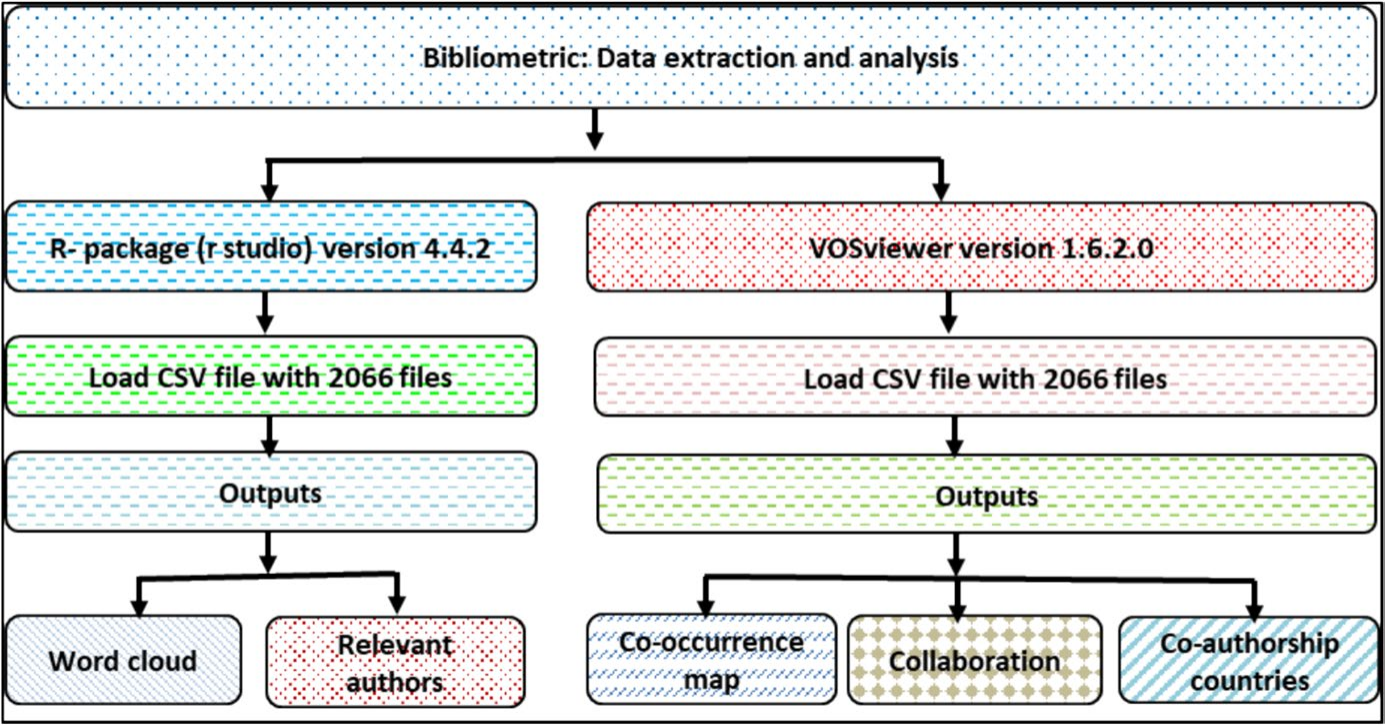
Flowchart illustrating bibliometric analysis methodology

As demonstrated in Fig. 5, the bibliometric data extraction and process of analysis was conducted by two softwares, i.e., R studio and VOSviewer, using CSV files with 2066 documents loaded for visualization. The output that was generated using the R package were as a word cloud, whereas the outputs from VOSviewer included co-occurrence maps, collaboration analysis, and co-authorship analyses by countries. For better understanding, the flowchart in Fig. 5 summarizes the sequence of loading the CSV data and corresponding outputs generated by each software tool. The results of this bibliometric analysis enable researchers to identify the correlations between the frequency of co-occurring keywords in the collected articles, co-occurring networks, annual scientific distribution, and authorship by country, as well as the essential future research directions for the themes in this discipline.

**Fig. 6:**
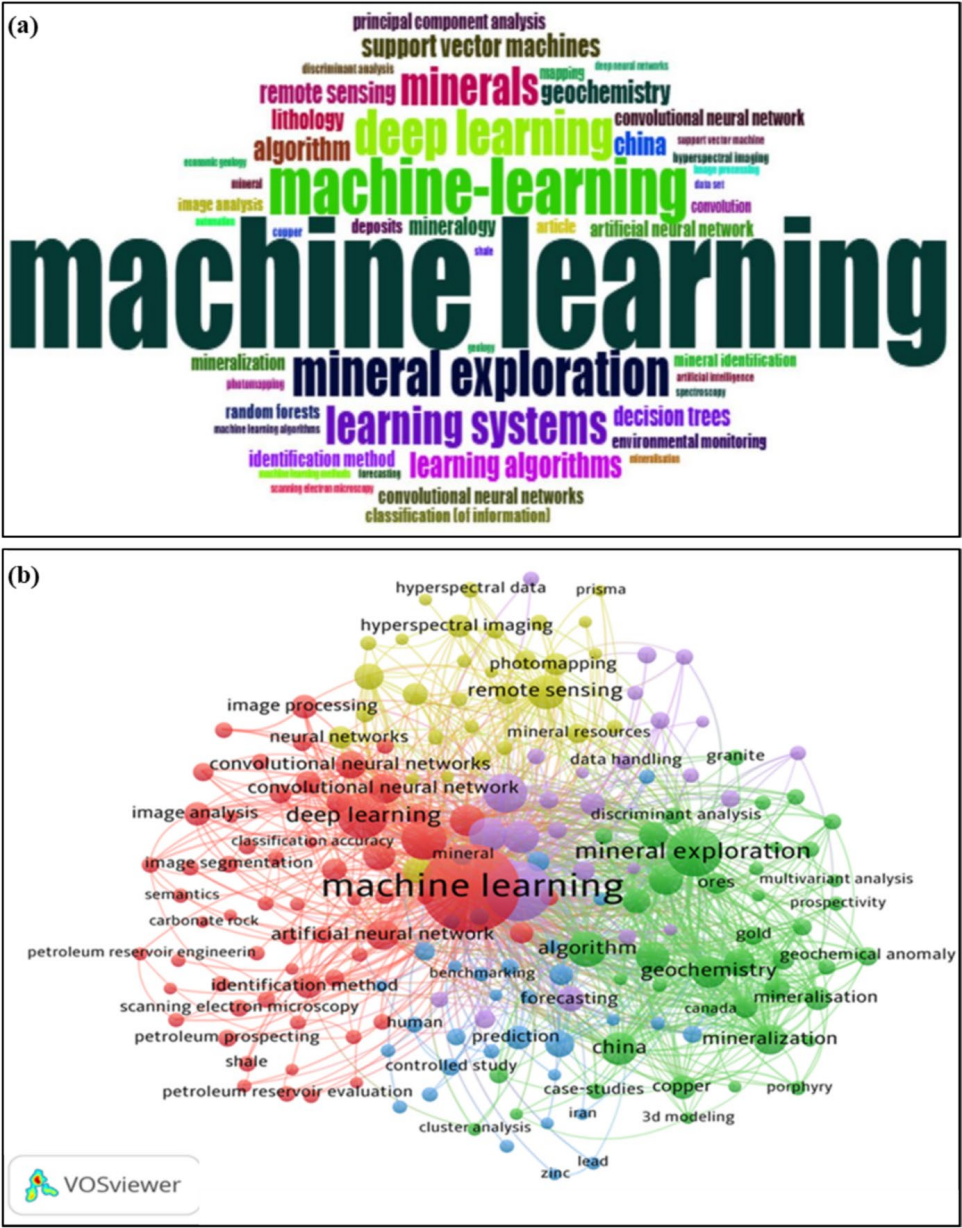
**a** Word cloud indicating the key words for artificial intelligence for the mining process and mineral characterization, and **b** co-occurrence network indicating the key words for artificial intelligence in mining processing and mineral characterization

The word cloud in Fig. 6a shows frequently used themes in keywords, titles, and abstracts in AI in the mining process and mineral characterization. The size of every word reveals its frequency or significance [15]. According to the size, words are ML, mineral exploration, and DL, learning algorithms are the most frequently searched. The less frequently searched words include ML algorithm tools (convolution neural network, DT, KNN, SVM), remote sensing, mineral identification, image segmentation, HSI, and mineral exploration. These words are in line with keyword searches and indicate the strong focus areas. The latter discovery implies that AI in the mining industry is still in its early stages globally. The word cloud visualization emphasizes research hotspots and trends which allow the fast identification of the emerging areas [19]. Co-authorship is a common and well-studied form of collaboration in science, and co-authorship network analysis can reveal an author's average publication and active years in a specific field of research [22, 23]. This visualization is useful for scholar and industry development on ML in the mining process and mineral characterization field which also allows the fast identification of the emerging areas [19]. The network map is shown in Fig. 6b, illustrating the interconnection and inter-relationship between the keywords.

The co-occurrence network map of keywords reveals four major clusters, indicated by five colors: red, yellow, green, blue, and purple, corresponding to ML, mineral identification, mineral exploration, GIS-remote sensing, and mineral deposits. These clusters comprise nodes that are interconnected by lines. Each cluster is attributed to the strength of the connections between keywords in the published documents. The analysis shows a strong connection with ML, mineral identification, and mineral exploration. Each node in the network represents distinct keywords, while the lines reflect the channels of association between them. The size of each node illustrates the frequency of usage for the corresponding keywords. Furthermore, the suitability cluster is associated with mineral exploration, image analysis, convolutional neural networks, artificial intelligence, algorithms, HSI, mapping, minerals, and segmentation. The mineral identification cluster relates to mineralization, mineral geochemistry, gold, and copper. Meanwhile, GIS-remote sensing is linked to HSI, mineral resources, data handling, and photomapping, which characterize GIS-remote sensing activities during mineral exploration. Notably, ML is strongly connected to deep neural networks, algorithms, bench marking, and semantic segmentation, which provide significant advantages for AI in the mining process and mineral characterization. This suggests that the utilization of AI in mining process and mineral processes is a key field of research for addressing mine safety, quality control, and accurate mineral characterization. This network visualization map demonstrates the important opportunity for developing nations to focus on research niches, such as mineral beneficiation optimization AI-assisted analytical characterization and the context of mineral exploration challenges where great contributions can be made without solely relying on advanced computational resources.

**Fig. 7:**
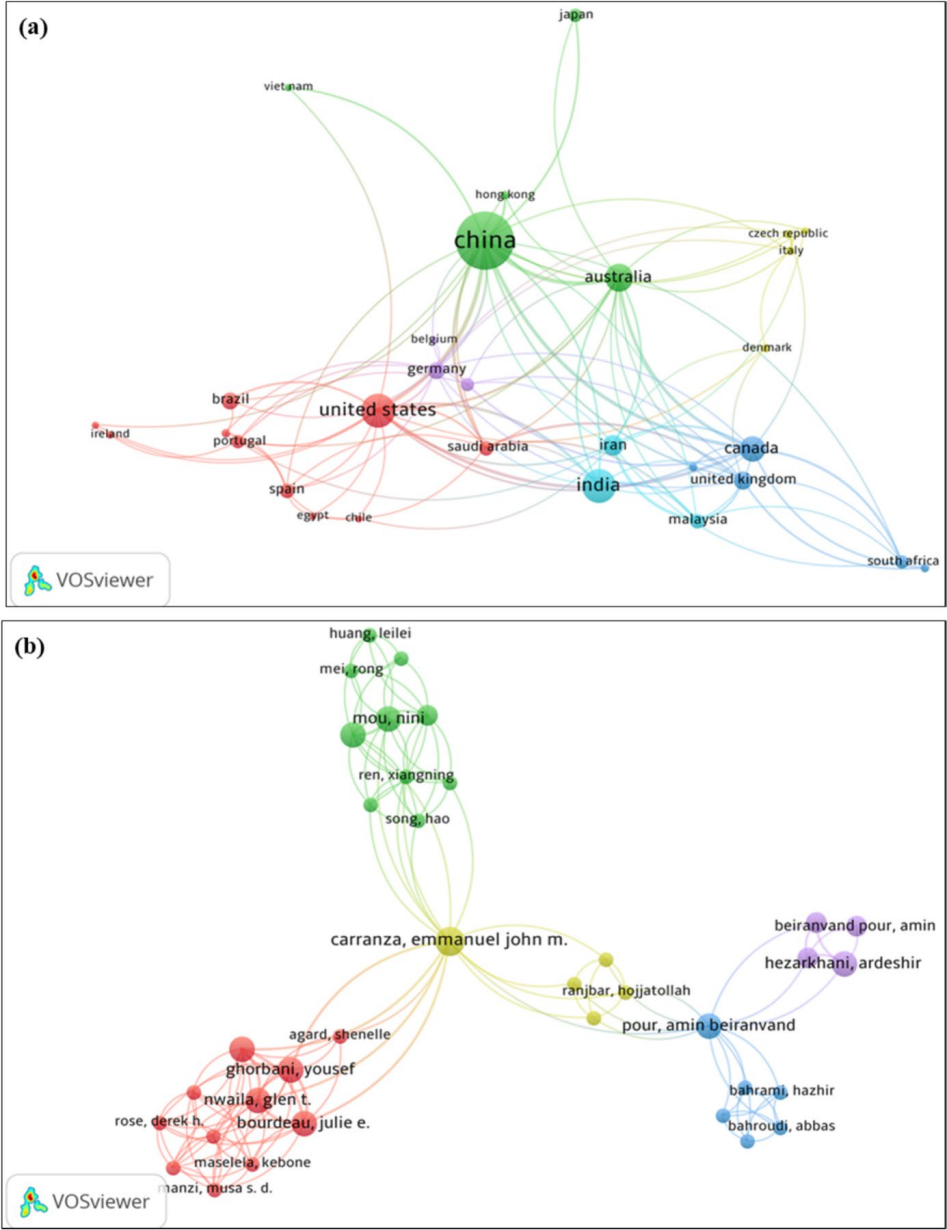
**a** Co-authorship map of authors and **b** co-authorship map of the most productive nations in ML in mining process and mineral characterization

The distribution of countries by region is shown in Fig. 7a. The closer two countries are to each other in VOSviewer, the stronger their relatedness and the more significant the link between them. The results of co-authorship indicate that China, the United States of America, and Australia are the most affiliated countries. China is linked to 53 countries/territories with 1780 citations, the United States of America has 41 links with 1260 citations, and Australia has 32 links with 563 citations. As illustrated in Fig. 7b, China and the USA serve as central nodes in global research networks as they are the most interconnected countries. Interestingly, underdeveloped countries like South Africa show growing global interconnectivity, with a total link strength of 11 and 8 citations. In conclusion, the results from the co-authorship analysis by country indicate the maturation of international collaboration in the field of AI and robotics applied in mining processes and mineral characterization, demonstrating the importance of international partnerships in advancing the research field and highlighting technological gaps and expertise across various countries and regions. The size and width of the links (lines) indicate the intensity of the connection between authors, where the node size on a network map reflects relative frequency. Stronger connections are represented by larger node sizes and wider links, which appear most frequently in the network map. Figure 7b displays the co-authorship by authors retrieved using VOSviewer, showing the characteristic features of a robust co-operative network, indicating that research in the field of AI related to the mining process and mineral characterization has reached a global scale. The lack of interconnectivity observed for South Africa and Malaysia might be due to factors such as the dynamics of international collaboration, the diversity of research partners, the high percentage of foreign postgraduates and visiting scholars, and strong research funding. It is also important to have a flexible and stable research policy to ensure the sustainability of international collaboration. However, the existence of emerging linkages suggest the growing opportunities for knowledge dissemination, young researchers exchange program to strengthen international collaboration, which is crucial for enabling developing nations to access experts and training to develop AI-driven mining solutions, mineral characterization, and long-term research sustainability.

## Mining Value Chain with Emphasis on Mining Exploration

**Fig. 8:**
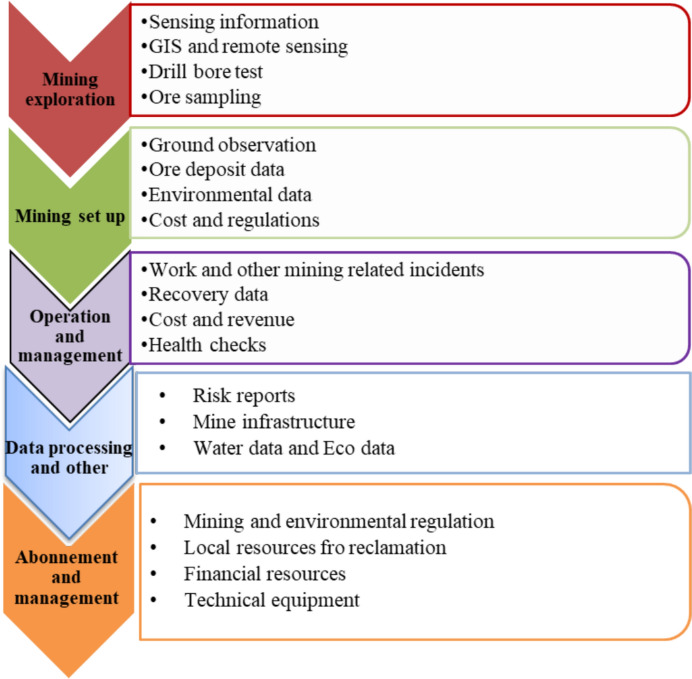
Schematic flowchart for the mining value chain

The mining value chain starts from exploration up to management of the abandoned mines or rehabilitation of the abandoned mines [25]. These processes are summarized in Fig. 8. Additionally, the mining value chain is a sequence of interconnected phases that transform mineral resources into marketable goods while meeting environmental and social responsibilities. The exploration involves geological surveys and remote sensing methods to find economically feasible mineral resources. The next step is to evaluate the resources and conduct feasibility studies to determine technical and financial viability. Mining development involves mining design, construction, and infrastructure setup. This section discusses the exploration and mining operation part of the value chain and environmental monitoring, safety protocols, and the exploration tools for rehabilitation of abandoned mines and effective post-mining land use [24]. Moreover, active mining involves extracting minerals, which necessitates careful management of resources and waste [25]. Modern technologies such as ML and AI are used to optimize operations, reduce costs, and increase recovery rates [1].

### Mineral Exploration Transformation

The mining value chain begins with the exploration process as the first stage and a crucial phase in the chain that assesses project feasibility through geophysical surveys [26]. In ancient days, mining and mineral exploration practice relied on direct observation for surface examination of crops, soils, and sediments, which was transformed into renaissance systemization (science-informed methods) which was invented and formalized by Georgia Agricola, a German physicist and Humanist, in the sixteenth century [27]. The twentieth century produced substantial advances in geophysical technology, geochemical sampling, and drilling techniques, which changed geological exploration [28]. There have been increasing developments in mineral exploration, adapting new methods including magnetic, gravitational, and seismic, remote sensing, and geological mapping to identify mineral deposits [28]. These later developments increased the precision and efficiency of resource identification and extraction, resulting in more sustainable practices in mining and hydrocarbon exploration. These innovations utilized the gravity survey, magnetic survey, and electromagnetic approaches, which are crucial in the identification of mineral ore deposits [29]. In 1990, a mineral deposit of Zn–Pb–Ag was located in Queensland by Thomas and co-workers using two regional gravity and ground magnetics [30]. Comprehensive sampling, geochemical assessment, and diamond drilling assessed the quality and quantity of the deposits, guiding development decisions and reducing financial risks [31]. Nagamani et al. evaluated the effectiveness of high-resolution data, which were produced by computer-aided GIS that was utilized between 1990 and 2012 by integrating the GIS with remote sensing [32]. GIS was invented in 1963 by Canadian scientist, Roger Tomlison [33]. GIS has become a leading digital tool for revolutionizing mining exploration by providing a comprehensive framework for data collection, analysis, and decision-making. Recently, the integration of AI and GIS-remote sensing technology has facilitated the identification of mineral deposits in a broader range, maximizing the efficiency, tracking changes over time, and predicting potential risks, thereby enabling more informed policy-making. Saadia et al. integrated geological data, geochemical data, and geophysical data into GIS and remote sensing to determine the spatial distribution of Pb–Zn–Fe–Ba mineral deposits in the Saharan Atlas [34]. Table 2 illustrates a summarized overview of the technological transformation in mineral exploration, and highlights the dominant focus and key technological contributions in each era. For better understanding, it also emphasizes the progressive integration of AL/ML (i.e., digital and automation tools), GIS, and remote sensing.

**Table 2:** Mineral exploration development

Period	Key exploration activities	References	Technological contribution
Georgia Agricola, 16th century	Renaissance systemization (transformation from artisanal to science-informed)	[35]	
19th century	Geological mapping	[36]	GIS introduction integration of spatial data and decision support
20th century	Geophysical technology Geochemical sampling Drilling technology	[28]	
1990s	Digital data and GIS for large surface mapping	[37]	Remote sensing for targeting minerals
2000s	Remote sensing	[32]	AI/ML-assisted GIS-based prospectively mapping
2010s	AI integration	[38]	
2020s	Robotics automation in mineral exploration	[39]	For application in real-time sensing and predictive analytics and decision-making

The following section discusses the specific GIS platforms that have played a critical role in mineral exploration.

### GIS Platforms for Mining Processes

Generally, GIS platforms aid in delineating mineralized zones, mapping artisanal mining activities for formalization and monitoring processes [40]. The subtopics below summarize and describe the utilization of various GIS platforms for mining processes and mineral characterization.

#### Geovia Surpac

**Fig. 9:**
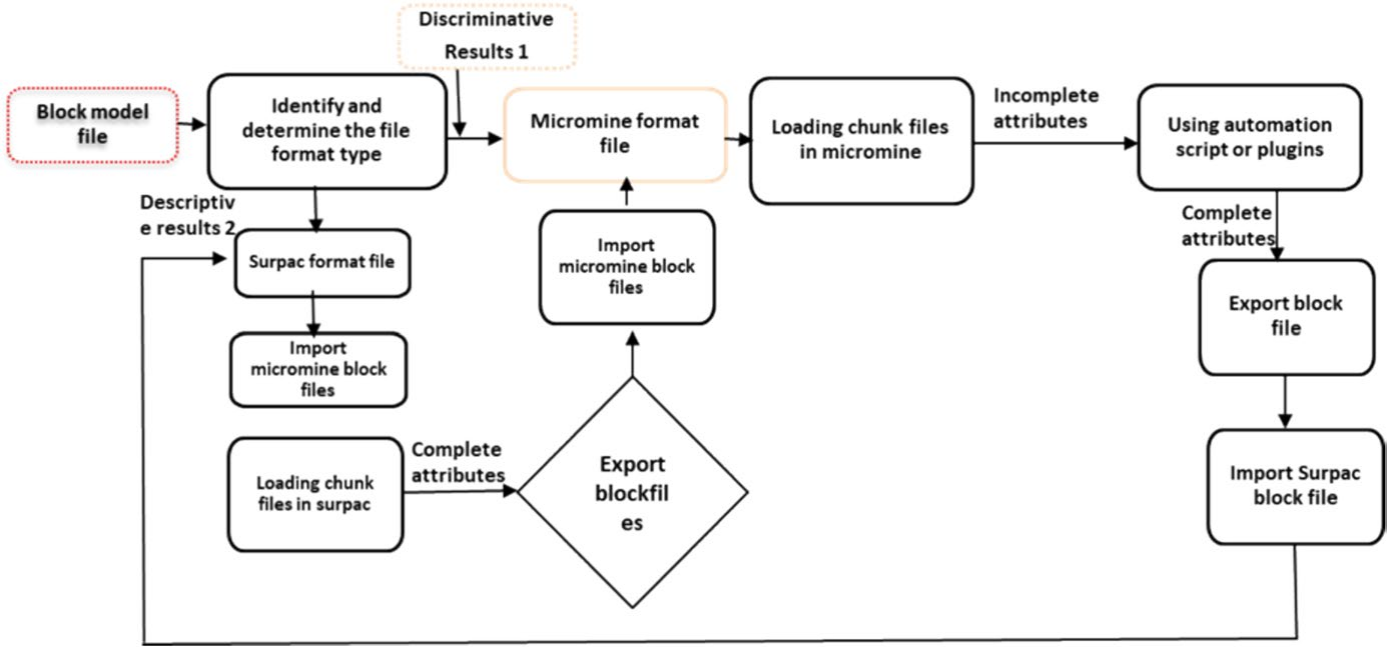
Schematic flowchart for Geovia Surpac, redrawn from [43]

Geovia Surpac was established by Dessaults Systems and is well known as one of the powerful software solutions for geological database administration and mine planning modeling software that excels at integrating mine planning and grade control [41, 42]. Its capabilities enable the production of precise geological models, which facilitate optimal resource estimation and operational planning. This GIS tool has attracted considerable attention due to its support for geological database management by enabling the geographic representation of boreholes and facilitating surface generation, which strengthens the visualization and analysis of data. In addition, it supports block modeling and estimation of resources through employing advanced block modeling techniques and allows effective data processing and visualization of reservoir qualities [41]. Moreover, the tool integrates mathematical algorithms for resource estimation, which is important for mining process optimization. Researchers from developed and developing nations have devoted research into the adoption of Surpac to support exploration, estimation of resources, mine design, and diverse geological environments. Over recent decades, an Australian mine company, Decian Gold Limited, has been a well-known Surpac specialist, using the software in complex mineral resource estimation [43]. Moreover, Ramos, i.e., the Hecla mine in the United States of America, utilized Surpac and Minesched software in underground and open-pit mine planning and cost estimation (budgeting). In most African mines, Surpac has been extensively integrated into critical minerals, including platinum group metals such as gold, as well as copper and other base metals. Kalamaba et al. from the Eurasian Resource Group, which is a major ore mining company in the Democratic Republic of Congo (DRC), integrated Surpac in mineral resource modeling and quality control. The Mopani Copper Mine specialized in underground mine design utilizing Surpac and MineSched [44]. Phalatse et al. employed Surpac and the Minex package in structural and quality estimation of coal resource modeling with an emphasis on the role of Surpac in integrating geospatial datasets and geological analysis, and in the interpretation of mineral resources [45]. The typical workflow of geological modeling and mine planning in Geovia Surpac is summarized in the schematic presented in Fig. 9.

#### ArcGIS

**Fig. 10:**
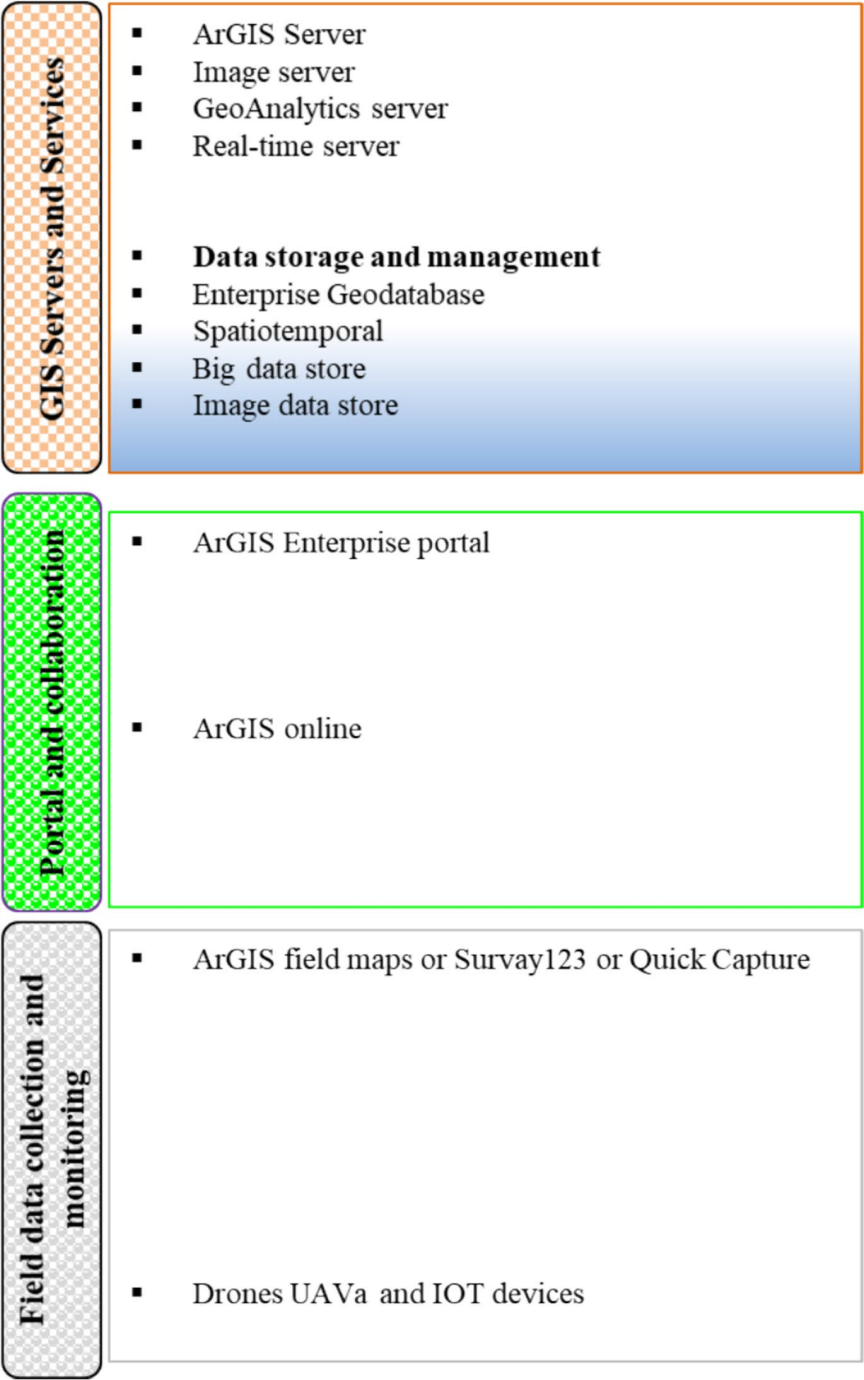
A flow chart display of key components and data flow in an ArcGIS-based mining application

ArcGIS, invented by Esri, is the flagship GIS software which plays a crucial role in mining exploration in both developed (Western countries) and developing nations (African) [34], with robust spatial analysis for data integration capabilities, having compatibility with remote sensing technology essential for modern mineral resource management. The South African mining and mineral councils, such as the Council for Geoscience, the Department of Mineral Resources and Energy, support ArcGIS for the national Geological Survey, mineral resource monitoring, and environmental assessment. In South African countries, Anglo American and Exxaro employ ArcGIS for hydrogeological studies, tailings risk management and terrain modeling, specifically in complex environments such as the Karroo (Cola deposits), Wits basin (Gold), and the Bushveld complex for PGMS exploration. Other African countries like Tanzania, Ghana, and the DRC employ ArGIS to integrate legacy geological data with satellite imagery for green field exploration and mine sites. Xie et al. integrated the SVM logarithm to create an extensive mineral proximity of copper–polymetallic deposits in the Katalag mining district using geochemical data, geophysical data, and multi-source geological data to solve the challenges associated with mining in deeper deposits [44]. As illustrated in Fig. 10, ArcGIS-based mining solutions start with data collection from field surveys, sensors, and remote sensing, then centralizes data storage in geodatabases. The GIS Server layer includes services for mapping, feature management, geographical analysis, and real-time monitoring. The application layer consists of desktop, web, and mobile interfaces that allow users to see, analyze, and manage mining data. Finally, the data are visualized using maps, dashboards, and reports, with collaborative sharing via ArcGIS Online or Enterprise platforms to support informed decision-making throughout mining operations. The GIS Server layer includes services for mapping, feature management, geographical analysis, and real-time monitoring.

#### Geomine and Datamine

**Fig. 11:**
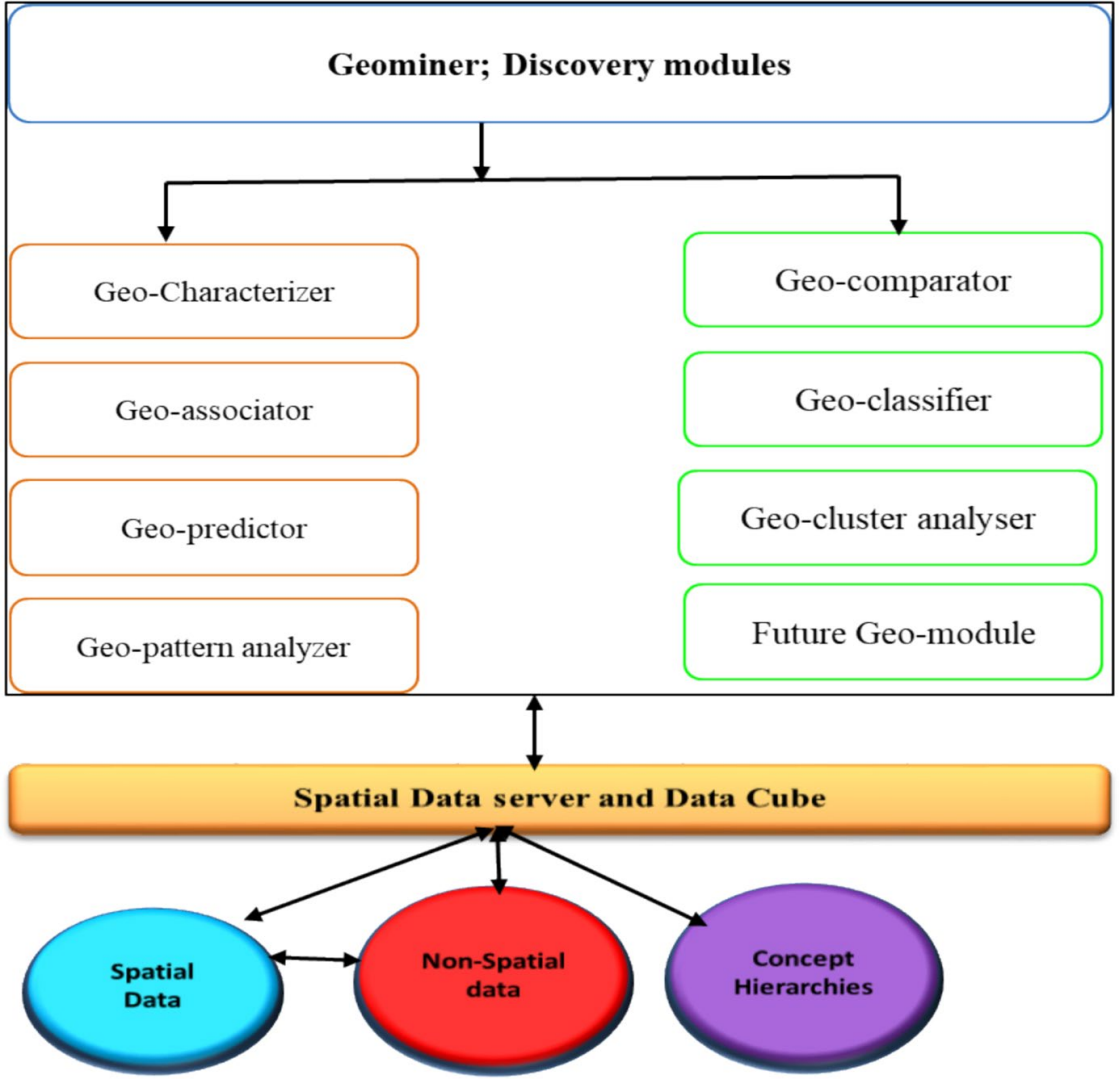
Flow chart for geological integration using Geominer

Geomine and Datamine are effective for modeling mineral deposits and have been successfully utilized in several mining industries, including the visualization of ore bodies and mineral resource estimation. The software mainly focuses on spatial data mining, allowing the extraction of high-level spatial information from large datasets that can be beneficial for identifying spatial data. Geominer is comprised of four components, geo-characterizer, geo-associator, geo-predictor and geo-classifier [46]. As summarized in Fig. 11, these modules consolidate geological data by pre-processing and integrating datasets such as drill-hole assays, lithology logs, and structural measurements, providing a uniform dataset, which is visually and statistically evaluated before being converted into a 3D model (Table 3).

**Table 3:** GIS platform analysis for mineral processes

GIS platform	Strengths	Weaknesses	Application
ArcPy	Advanced automation	High cost	Used for managing drill-hole databases, interpreting geochemical patterns and supporting the land use compliance
QGIS	Open-source	Limited support	Academic research
KVV	Real-time processing	Specialized	Underground mapping
Geomine or Datamine	Designed for mine and geological planning	Requires intensive training, and is less flexible for non-mining GIS needs	In the mining industry, geological modeling and effective for optimizing production schedules through block model regularization
Geovia Surpac	Geological database and modeling features. Strong on integration with mine planning and grade control It allows for the production of complete geological databases by combining borehole data, which includes coordinates and constituent grades	Not appropriate for broad land use GIS and broad environmental studies	Used for open-pit and underground mine design, mineral exploration and mine planning. For borehole development
Micromine	A user-friendly interface which include 3D visualization (through visual explorer) that is effective for geological modeling and resource estimation	Not widely used due to commercial licencing which limits its general functionality	Used for mine planning, mineral exploration, and drill-hole data management
Gocad mira analysis	Applies the GOCAD Mining Suite which has sophisticated 3D geological modeling with strong integration of geophysical and geochemical data	Drawbacks are high cost and steep learning curve	Used in complex geological modeling, geophysical interpretation, mineral system analysis, and uncertainty-driven resource modeling

### Machine Learning and AI in GIS and Remote Sensing

**Fig. 12:**
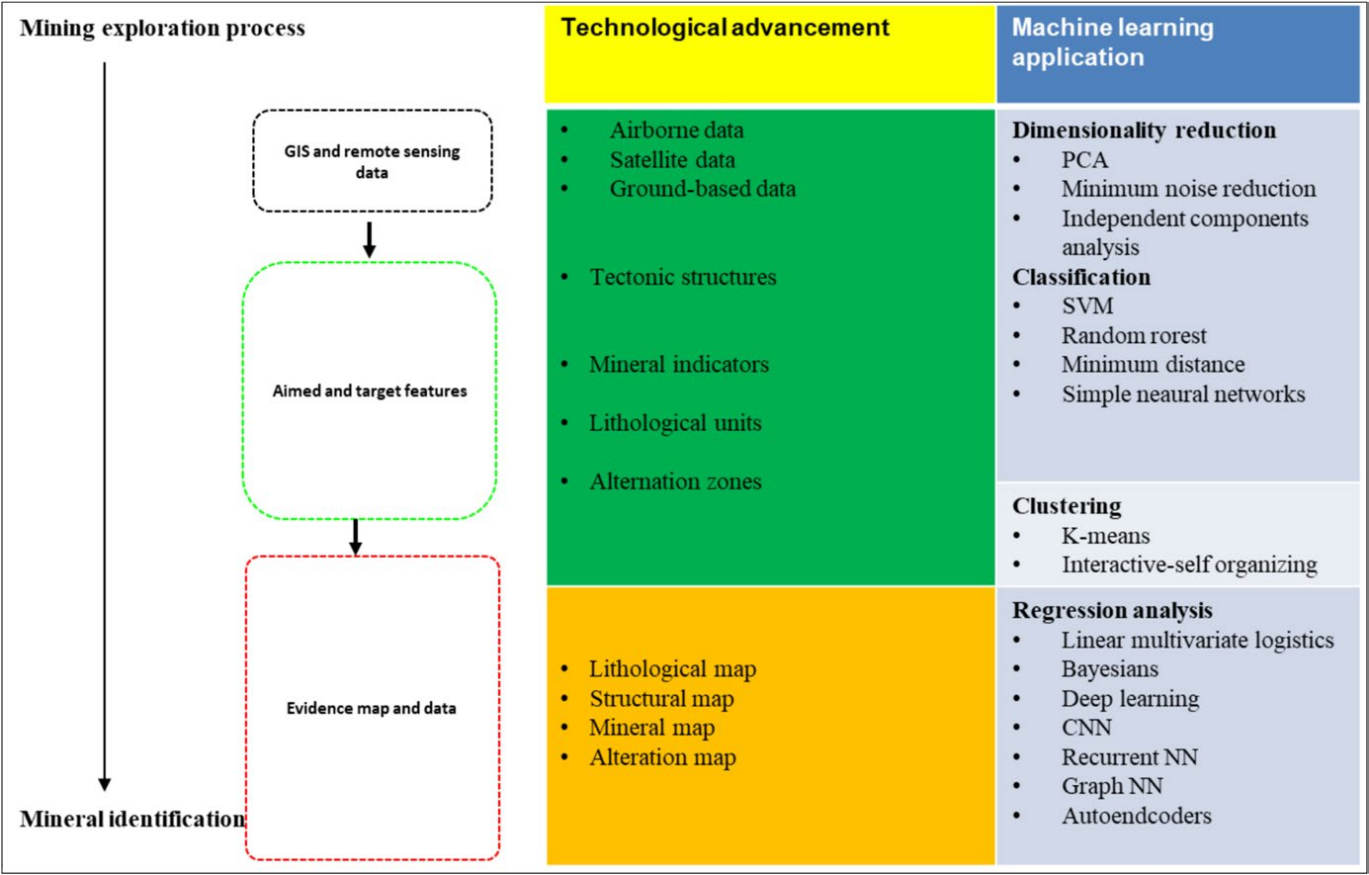
A flowchart summarizing the integration of ML tools in mining exploration

ML and AI algorithm integration to GIS and remote sensing have been transformative in the mining sector [47]. Furthermore, the emerging trends of ML and AI in GIS and remote sensing for mining processes have substantially improved mining exploration (i.e., enhanced data analysis, environmental monitoring, and mineral exploration facilitation) [48]. Kopec et al. investigated the environmental effects of underground hard coal mining in Poland using GIS tools such as spectral index, normalized difference vegetation index, geographical weighted regression, spatial radar inference, and ML techniques (i.e., random forest, RF). Shirmad and co-workers discussed the mapping of geological features in targeting ore deposits through emphasizing the role of ML in processing the data from remote sensing and its efficiency in noise handling and uncertainties [37]. The complex data have been considered using the DL approach for simplifying the data from remote sensing. The incorporation of DL, specifically CNNs, into GIS and remote sensing has greatly improved data analysis capabilities. This development enables more accurate geographical data interpretation, addressing issues such as large dimensionality and complicated patterns [49]. Li et al. reported the integration of DL in remote sensing by the state-of-the-art DL solutions such as image (pixel level) fusion, multimodal image and spatiotemporal [50]. Afrosheh and Askari explored the fusing of DL with remote sensing image analysis using a CNN algorithm and a short-term memory network with GIS to improve the efficiency of spatial data analysis and to address the shortcomings associated with complex patterns and high dimensionality [49]. Wen and co-workers explored spatial decision support systems with AI (automated ML) [38]. Figure 12 summarizes the integration of ML tools in mining exploration and advancement activities, including technology development and geological features up to mineral identification.

### Advantages of Using GIS and Remote Sensing in Mining Activities

GIS in mining activities supports mine planning, optimizes mine operations, and analyzes the environmental impact on the mining sites. GIS is a powerful tool for mine planning, optimization, and environmental impact analysis, allowing for effective management of mine data in georeferenced, spatial, and attribute formats, such as drill holes, ore bodies, mineral deposits, pit boundaries, and underground haulage roads. GIS also supports decision-making during mining development stages, such as ore reserve detection and estimation, visualization of uncertainties, and land-use selection after mine closure. It offers flexibility in multi-parameter considerations, such as optimal truck haulage route determination and in-pit storm water response design. GIS also reduces the time and effort required for detailed site investigation, allowing investigators to focus on mine reclamation planning without accessing undeveloped, dangerous, or hazardous areas [51]. This advancement enhances operational efficiency and minimizes environmental impact by enabling more accurate targeting of exploration efforts. Consequently, mining companies can improve resource extraction while adhering to sustainability protocols.

### Modern Exploration Process

Advanced technologies, including AI and ML, enhance accuracy, streamline operations, and reduce environmental effects and health risks. This strategic, data-driven approach guarantees sustainable resource extraction, efficiently fulfilling global mineral requirements [52]. The mining industry persists in innovating within this framework, and they enhance their proficiency in making informed decisions that maximize profitability while emphasizing sustainability. Investing in these technologies enables the mining sector to foster a more sustainable future, effectively balancing economic requirements with environmental responsibility. Recent improvements include AI-driven target generation and HSI, which have increased discovery rates by 30% and reduced environmental impacts [53]. The Bushveld Complex in South Africa illustrates how systematic exploration has expanded platinum reserves over the past two decades via extensive 3D modeling [54]. Table 4 summarizes the exploration methods, highlighting various sites and technology utilized.

**Table 4:** Comparison of modern exploration methods for new operating mines

Method	Greenfield sites	Brownfield sites	Technology used	Cost (USD/km^2^)	References
Airborne survey	Primary method	Verification	Magnetics/EM	5000–15,000	[55]
Soil sampling	Regional grids	Infill patterns	Portable XRF	500–2000	[55]
Drilling	Wide spacing	Tight spacing	Diamond/core	50–200/m	[55]
Remote sensing	Landsat/sentinel satellites	UAV/drones	Hyperspectral	200–1000	[55]

BHP's Olympic Dam expansion utilized subterranean drilling to increase copper reserves by 2.5 million tonnes, demonstrating the effectiveness of leveraging existing sites for resource augmentation [55]. This strategy reduces costs and mitigates environmental impacts, rendering it a favored approach for companies aiming to sustainably enhance their operational efficiency. This strategy utilized 4 billion tonnes of resources, thereby substantially advancing the sustainability objectives within the mining industry. Through the optimization of resource extraction practices and the minimization of environmental and social disruptions, companies could engage in a more responsible and sustainable approach to meeting global demand. This strategic alignment not only addresses the urgent need for responsible resource management but also enhances corporate accountability in the face of rising consumer expectations for ethical sourcing and environmental stewardship.

### Machine Learning in Drilling and Blasting

The conventional methods for drilling and blasting mostly face shortcomings related to operational parameters and variability in geological structures and conditions [56]. These approaches include cable drilling, auger drilling, rotatory drilling, diamond drilling, and directional drilling [57]. To address these bottlenecks, the advances of AI and ML provide promising solutions to optimize the drilling and blasting processes by allowing real-time monitoring and assessments, predictive analytics, and adaptive controls. ML is being used in mining, drilling, and blasting operations to improve safety, productivity, and cost-effectiveness. ML, by employing advanced algorithms, may optimize blast designs, forecast rock fragmentation, and improve decision-making in difficult mining scenarios [58]. There have been increasing attempts to integrate ML to enhance productive and operational efficiency. Munagala et al. used ML in reviewing drilling costs and blasting costs with 89% and 23% reductions in costs, respectively, with emphasis on the need for the standardization of data and evaluation metrics to enhance operational safety and effectiveness [59]. The prediction of rock fragmentation parameters such as spacing, powder factor, and burden by Gebretsadic and co-workers used ML algorithms (like SVM, RF regression, neural network regression) [58]. Kokkinins et al. reported the advancement of mining technology and real-time monitoring through integration of ML and drilling data, highlighting AI and the Internet of Things (IoT) and robotics to improve mining safety and efficiency [57].

## Conventional Mining to Modern Mining

**Fig. 13:**
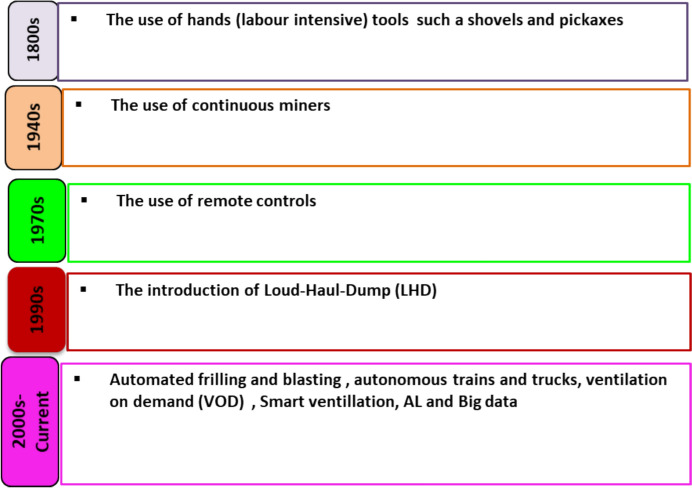
Schematic flowchart of mining evolution technology from the 1800s

The evolution of conventional mining operations began from the 2nd–3rd industrial revolutions, displaying significant technological developments that transformed the mining operation and mining extraction processes [6]. As displayed in Fig. 12, generally during the 2nd industrial revolution (between 1850 and 1940), industrialization was dominated by labor-intensive (manual) operations and steam-powered machinery. Technological advancement, electrification, and early computing technology occurred in 1950–2000, referred to as the 3rd industrial revolution, enhancing the efficiency and safety in various industrial processes, including mining [60]. The mining operation during the 2nd industrial revolution used steam-powered engines to enable deeper and effective mineral extraction; however manual labor was still applied in hazardous areas where machines could not reach. The mining evolution since the 1800s has been summarized in a flowchart, shown in Fig. 13.

### Robotic Automation Systems in the Mining Process

**Fig. 14:**
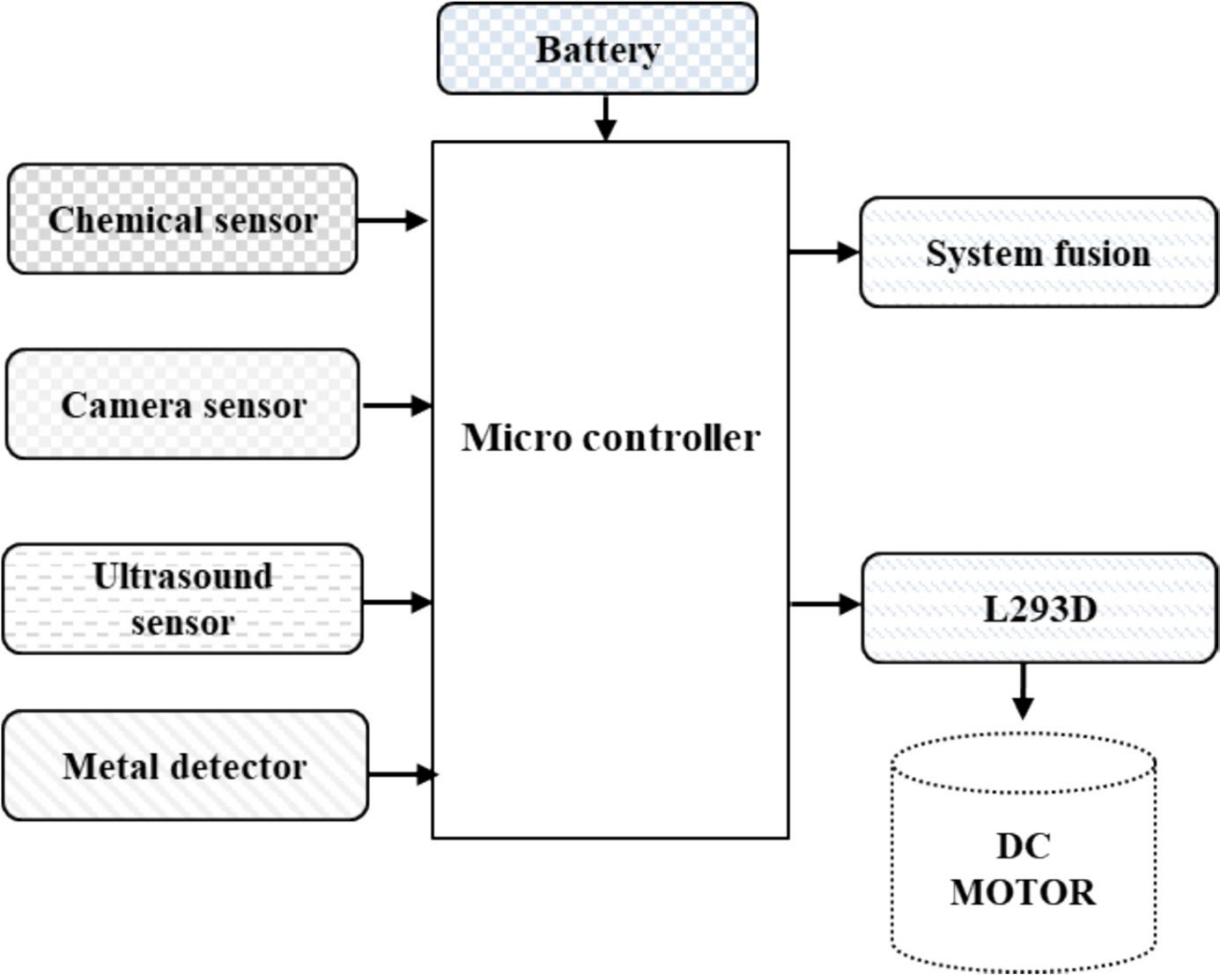
Flowchart of the block diagram for a mining robot

The advancements of robotics in the mining sector and the increase in robot use have created a demand for computer simulation of robots, among the goals of which are the design of new robots, task planning of current robots, performance evaluation, and cycle time estimation [39]. The continuous improvement in robotic autonomous systems (RAS) has created new opportunities in the mining sector, with the potential to replace people in numerous roles [61]. Moreover, RAS is being investigated to enhance the safety (i.e., hazardous environments, labor shortage) and efficiency in mining operations [39]. The commonly used robotic systems utilize a configuration that encompasses a microcontroller-based architecture that is integrated with an ultrasound sensor, a camera module, and supported by system fusion (sensor fusion algorithms) for robust decision-making. Figure 14 summarizes the typical flowchart, synthesizing insights from current advances in mining robotics utilized in underground sensing technologies (Table 5).

**Table 5:** Robotic systems overview by leading mining companies and their application in enhancing efficiency and minimizing risks

Company	Robotic system	Commodity mined	Application	References
Sandvik	Autonomous truck	Gold, iron, and copper ores	High-accuracy drilling and haulage reduce human exposure to hazardous areas	[62]
Komatsu	Frontrunner autonomous haulage systems	Coal, copper, and iron ores	Autonomous haulage systems to improve operational safety and productivity	[63]
Rio Tinto	Autonomous drill	Iron, bauxite (Al_2_O_3_·2H_2_O), and copper ores	Accuracy in drilling and blasting while minimizing operator involvement in hazardous environments	[6, 57]
BHP Billiton	Autonomous rail (AutoHaul)	Iron ore	Applies the driver's train system to eliminate human error in long-distance ore transport	[64]
Anglo-American	Spot robot	Platinum, diamonds and copper ore	Applies for site inspection in hazardous zones and to minimize risk to human inspectors	[65]
Vale	Robot inspection drones	Nickel and iron ores	Assists with remote inspection of tailings dams and confined spaces to prevent exposure to hazardous conditions	[66]
Barrick Gold	Semi-autonomous loaders		Applied for remote-controlled loaders in underground operations reduces risk to operators	[67]

## Conventional Characterization to Current AI-Assisted Analytical Characterization of Minerals

The research of analytical characterization of mineral has progressed from purely manual to analytical techniques based on current AI-assisted autonomous systems. The ancient traditional approaches were dominated by laboratory-based techniques like XRD and XRF, used for crystallographic phase and bulk elemental composition, respectively [68]. Other instrumental techniques used for bulk elemental composition are, e.g., ICP-OES, ICP-MS, AAS, and SEM–EDX [69]. These techniques offer high precision, accuracy, and high throughput. However, they are expensive, and rely on experts for interpretation and chemometric tools and long times for interpretation. Analytical characterization workflow for the characterization of minerals has integrated/migrated to chemometric tools, semi-automated sample preparation, and digital data. In early 2000, instrumentation techniques like QEMSCAN and mineral liberation analyzers automated mineral quantification while Rietveld refinement enhanced XRD spectra analysis [70]. This introduction of ML in 2010 has also gained interest for applications in spectral and imaging datasets for identification of mineral phases and mineral mapping, specifically in SEM–EDS and HIS [71].

**Fig. 15:**
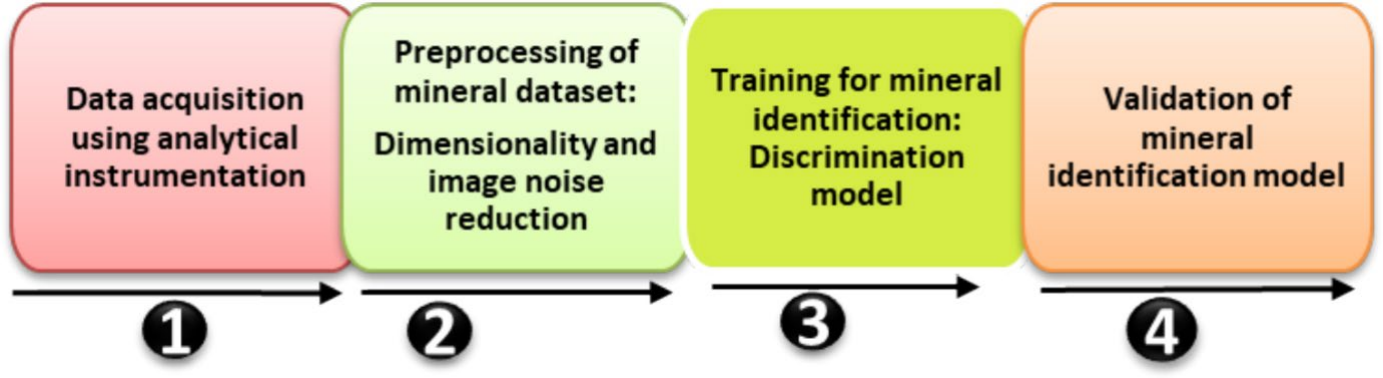
Schematic of the basic process of intelligent mineral identification

Research development attempts have been based on the enhancement of accuracy, high throughput, and reproducibility within the specific silos, limiting cross-technical data integration [72]. In the past 10 years, the application of ML algorithms emerged, based on neural networks (i.e., ANN, CNN, k-NN, DT), and trained to categorize 3989.34 spectra of minerals, automation image interpretation from SEM–EDS and LIBS, and to identify the spectral patterns in HS datasets [73]. Additionally, ML algorithms allow for the provision of consistent results that are reliable and reduce laboratory analyst biases. However, these AI approaches are inhibited by the quality and representativeness of the training datasets. Recently, around 2020, the deployment of DL models was witnessed to enable real-time mineral characterizations [74]. Through the utilization of DL models, the datasets from XRF, XRD, and HSI can be combined into integrated datasets for geochemistry exploration. The extensive research has shifted the focus in the future generation and establishment of autonomous ML intelligence and quantum ML. Figure 8 summarizes the roadmap for the analytical characterization of minerals. The common analytical techniques used for mineral characterization are discussed in the following subsection (Fig. 15).

Large and complex datasets are generated by analytical instrumentations comprising information which is crucial for analytical chemists in mineral characterization [75]. There are difficulties in measuring minerals chemically utilizing the large amounts of complicated data that are produced by modern instrumental techniques, as these data frequently contain instrumental artefacts such as noise, batch effects, baseline shifts, signal overlaps, and discrepancies from various instruments [9]. These shortcomings necessitate the processing of data using advanced processing tools like AI. AI has emerged as a transformative tool, offering advanced capabilities in pattern recognition, data integration, and automated decision-making. AI in analytical characterization of minerals incorporates advanced computer approaches to improve mineral identification, processing, and exploration of minerals [19]. This entails analyzing datasets using AI methods like ML models, neural networks, and genetic algorithms, to improve accuracy, and to automate mineral identification. These are popular for classifying tasks in mineral identification and identifying patterns. In order to increase process efficiency, they help automate the analysis of mineralogical data [19, 76]. When using geochemical and geophysical data, AI, and more especially ML models, are utilized to enhance knowledge of rock types and mineral resources [77]. ML neural network techniques like ANN and CNN have been employed to classify minerals from microscopic images, achieving accuracy rates above 90%, by simplifying mineral identification tasks, surpassing human assessors, and being relevant to a variety of difficulties like exploration and environmental management, AI improves the efficiency, accuracy, and application in geological research, but issues like model training and data quality still exist [19, 78].

### Basic Process of Artificial Intelligence in Analytical Mineral Characterization

**Fig. 16:**
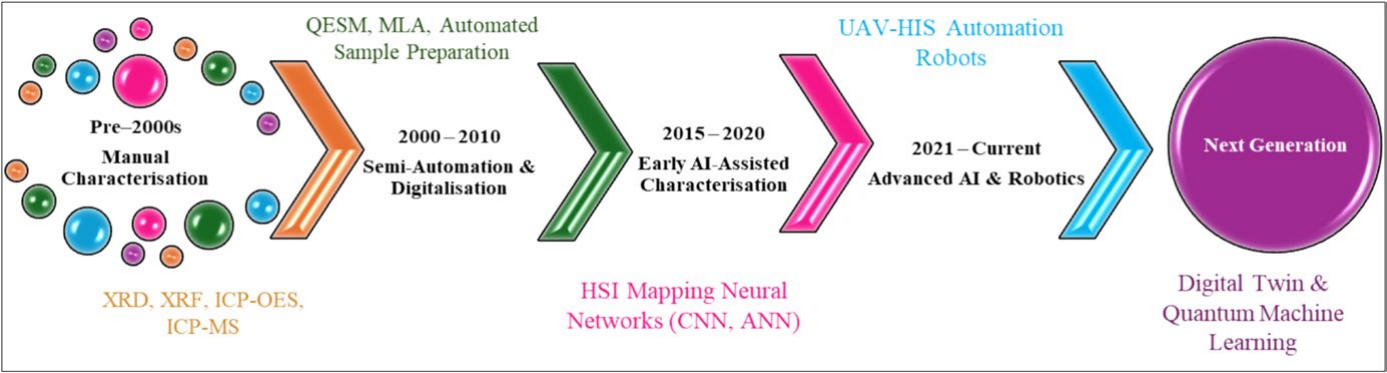
Schematic of the analytical characterization evolution from the traditional laboratory-based approach to modern AI-assisted and autonomous methods

AI and robotics are becoming recognized as transformative techniques for mineral characterization which improve efficiency and safety in mining operations. The integration of these technologies enables advanced mineral identification, processing, and extraction, resulting in a dramatic shift in traditional mining techniques. These ML and DL tools, such as KNN, DT, PCA, and ANN, are utilized for the classification of minerals and visualization [19]. As illustrated in Fig. 14, the basic process of identifying minerals is consistent across all characterization approaches, and these processes can be summarized according to four different stages: (1) acquisition of mineral datasets; the analytical instrumentation is being used to acquire data on minerals under study, including physical data, graphics, and images in the case of SEM analysis; (2) includes the pre-processing of mineral datasets based on dimensionality and image noise reduction to improve the accuracy of the mineral classification in the following step, (3) training the model for mineral identification, where AI is used to train the discrimination model for mineral identification and (4) validate the accuracy of the mineral identification model, which is used to distinguish between the data to be recognized and determine the mineral category. Different discriminative approaches are utilized depending on the mineral datasets. The present discriminative models are also diverse, and the accuracy rate varies significantly. In spite of the short-term development of AI, it has been widely used in the exploration of intelligent mineral identification, and crucial milestones have been achieved (Fig. 16; Table 6).

**Table 6:** Application and significance of AI in analytical mineral characterization

Analytical method	Mineral analysis	ML algorithms	Description	Significance	References
HSI	Fundamental scanning to determine the alteration zone of porphyry copper deposits	SVM (supervised) CNN (DL) PCA DEEP belief network	AI allows pixel-wise mineral mapping utilizing spectral–spatial data	Uses high-resolution mineral mapping and remote sensing to produce high-throughput and spatial data that is AI-adaptable. Non-linear mineral pattern recognition. Recognizes six types of minerals with up to 91% accuracy on unseen images, indicating high efficiency for mineral classification	[79] [80]
		Random forest (supervised)	Multi-source mineral data, i.e., spectra geochem	Effective for hyperspectral data classification and obtaining high accuracy in mineral mapping	[81]
LIBS	For lithological mapping for identification of mineral and quantification of critical elements like REEs and Li-bearing and most elements (trace and major)	PCA + ML (unsupervised and supervised), CNN, DT	ML logarithm correct spectral interferences and mineral identification from plasma emission at real-time elemental profiling and classification of rocks in mineral exploration	Induces high-speed analysis with less time in sample preparation and also compatible for drone platform	[82]
XRF	Mineral composition and elemental concentration determination of mineral, targeting medium to major elements like Si, iron, copper, and gold ores	Autocoder neural network, CNN	AI tool interprets spectra and elemental quantification in mineral ores	Very fast in situ analysis and non-destructive suitable for analysis of ore grade and composition	[83]
SEM		PCA (unsupervised and supervised)		Dimensionality reduction before classification. PCA is effective for summarizing complex datasets such as those from SEM, facilitating the best mineral classification	[84]
		CNN (DL)		Image-based mineral identification	[78]

#### Machine Learning Tools in LIBS

**Fig. 17:**
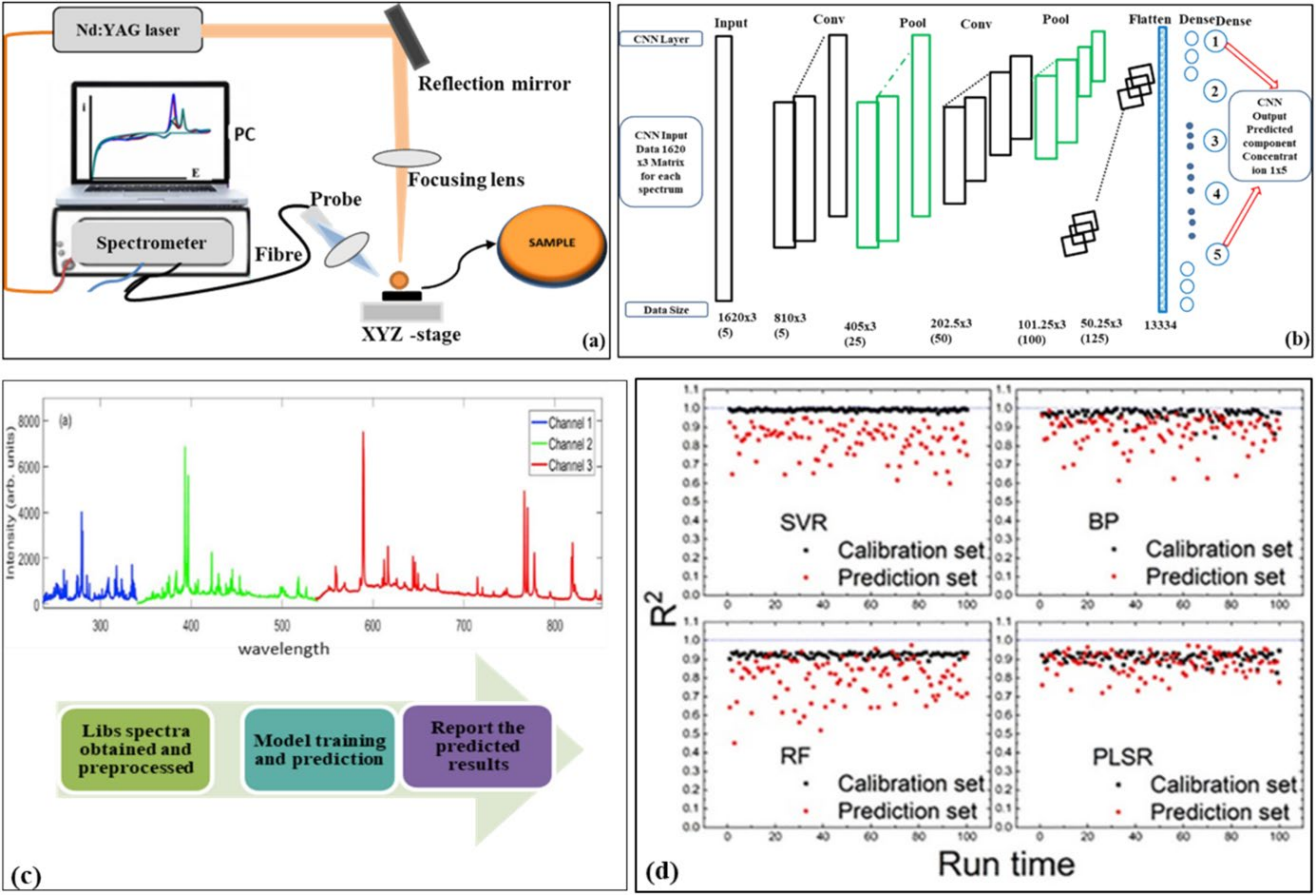
**a** Schematic of LIBS experimental setup [88] and **b** LIB-based CNN topology and relevant parameters of main layers (i.e., **top** 3 convolutional layers, 3 pooling layers, 1 flattening layers, 2 dense layers) in CNN, **bottom** denoting output data size of the corresponding layer, **c** LIBS spectrum comprising three spectral segments/channels, and **d** prediction results of different calibration models [10, 89].

ML plays a crucial role in LIBS, addressing limitations through data pre-processing, feature extraction, dimensionality reduction, noise reduction, classification, quantitative analysis, automated detection, and multivariate analysis. ML algorithms like SVM, RF, and neural networks provide high accuracy in identifying material types, predicting elemental concentrations, and automating detection [78, 85]. The sophisticated atomic emission spectroscopy method known as LIBS for the rapid, in situ elemental analysis of solid, liquid, or gaseous materials serves as a powerful analytical instrument. It directs a high-energy laser beam onto a sample's surface, causing a little section of the sample to be ablated and a micro-plasma to emerge [19]. The composition of the sample can be determined by analyzing the light that the plasma produces, which contains spectral lines that match the components in the sample [86]. Meima and co-workers discussed the utilization of LIBS for studying the Bushveld complex, South Africa (i.e. Upper group 2, and Merensky) mineralogy (mineral chemistry), such as plagioclase, chromite, and orthopyroxene, to provide spatially resolved chemical analysis data, describing the mineral chemistry variation and mineral partitioning while highlighting rock texture features [87]. However, LIBS suffers from challenges associated with the difficulties in achieving ideal accuracy for quantification of the multi-chemical components in a target geological sample [88]. The traditional LIBS analysis setup is displayed in Fig. 17a, which includes the laser, optical systems, 3D translation stage, spectrometer, and PC (i.e., computer). As indicated, the optical system mainly focuses the laser on the surface of a sample, while ensuring measurement effectiveness and ablation on undetected areas. The displayed spectra (i.e., output signal) are captured by optical fibers. The ML algorithms like CNN and deep CNN assist LIBS for multi-elemental quantification. Li et al. reported more than 1400 spectra of LIBS collected from China national standard reference materials by evaluating the root mean square error value of the estimation [12]. The results indicated that the CNN-based approach is a potential algorithm for LIBS quantitative quantification with high efficiency and accuracy. Figure 17b summarizes the CNN-LIBS architecture for analysis.

Liu and co-workers reported the integration of ML and spectroscopy techniques with the main focus on the LIBS spectra modeling approach for coal identification, real-time monitoring, quality evaluation of calorific value, and volatile matter.

Harefa et al. demonstrated that the combination of nonlinear manifold learning and multivariate analysis has the potential to classify samples based on LIBS with reasonable accuracy. The prediction labels indicated that the Isomap-SVM model achieved the best classification performance, with classification accuracy, the number of dimensions, and the number of nearest neighbors being 96.67%, 11, and 18, respectively. According to Harefa et al., recent advancements in multivariate analysis as classifiers have helped interpret datasets from various LIBS applications [90]. For instance, when using an RF algorithm as a classifier for iron ore classification, Sheng et al. achieved accuracies of 98.50% for the training set and 96.00% for the test set [91]. Zhao et al. used an SVM to identify the geographical origins of honey, multi-floral honey, and acacia honey, and found that SVM performed satisfactorily [92].

Finally, Sheng et al. pointed out that SVMs and RF ML methods were evaluated comparatively on their ability to predict unknown iron ore samples using models constructed from a predetermined training set. Although the results showed that the prediction accuracies of SVM and RF models were acceptable, RF exhibited better predictions of the classification. The study presented here demonstrates that LIBS–RF is a valuable technique for the identification and discrimination of iron ore samples, and is promising for automatic real-time, fast, reliable, and robust measurements [91].

#### Machine Learning in XRF Analysis

XRF is regarded as a powerful technique for analyzing solid samples and has been widely used since the 1970s for bulk elemental analysis of geological samples, providing the elemental concentration of elements in pressed pellets and fused Li-borate beads comprising rare-earth elements (REEs) [93, 94]. XRF is categorized into WDXRF and EDXRF which are used for the determination of mineral composition and elemental concentrations [95]. However, the challenges associated with XRF include the high average limit of detection and high errors at concentrations between 1 and 10 µg/g [93]. ML has the potential to expand the capabilities of XRF, including the identification of elements at lower LOD and predicting the properties of geological samples that do not have characteristic features of K, M, and L emission lines to improve the efficiency and accuracy [83]. Andric et al. utilized DL based on autocoder neural networks as a tool for dimension reductions of data collected from EDXRF spectra. The results allowed the best possible reconstruction of the original EDXRF spectrum and informative extraction features used for size reduction.

#### Machine Learning in ICP-OES Analysis

ICP-OES has emerged as a powerful and essential analytical technique for multi-elemental analysis across various fields due to its rapid analysis, high sensitivity, and ability to handle multiple sample matrices [89]. Previous research has shown that ML is increasingly integrated into ICP-OES to improve the efficiency and accuracy of the analytical results [90]. This integration enables real-time monitoring of matrix effects and prediction of analyte element recoveries, which significantly increases the reliability of the results [91]. ML has become increasingly popular in various sectors, particularly in analytical chemistry, over the last decade. In ML, supervised learning methods such as partial least squares regression and generalized linear models have demonstrated high predictive accuracy, with *R*^2^ > 0.9 for the percentage recoveries of elements like cadmium (Cd), cobalt (Co), chromium (Cr), and lead (Pb) [92]. Carter et al. reported on the estimation of matrix severity (carbon), evaluation of its effects, and the prediction of analyte (Na, Ca, Na + C, and Ca + C) recovery in ICP-OES using both supervised and unsupervised ML tools [92].

#### Machine Learning in ICP-MS Analysis

The integration of ML with tools and ICP-MS has grown significantly, increasing markedly in enhancing calibration, data analysis, and applications across various fields, such as clinical research and mineral characterization [90, 93, 94]. The ML tools address the complexities of ICP-MS operation, optimize its performance, and improve the interpretation of trace element data. This application of ML to ICP-MS integration has opened new frontiers in trace element analysis, especially for complex environmental, biological, and geological matrices [95]. Manual calibration and matrix corrections are often necessary for interpreting such high-dimensional data, which can be labor-intensive and prone to systematic errors [96]. ML algorithms, including PCA, SVM, DT, and neural networks, can uncover hidden patterns, correct matrix effects, and enhance signal interpretation, thereby improving the ML algorithms, streamlining this process and boosting analytical efficiency and precision [97].

Supervised learning models are especially useful for developing predictive tools for sample classification, contamination source apportionment, and quantification of trace elements, even in cases of signal overlap or interference [98]. For example, regression models trained on spectral characteristics can be used to train regression models to estimate target analyte concentrations without removing the need for extensive calibration [99]. Forensic geochemistry and environmental monitoring greatly benefit from visualizing and grouping samples based on compositional similarities facilitated by unsupervised learning techniques, such as clustering and dimensionality reduction, e.g., t-distributed stochastic neighbor embedding (t-SNE) and PCA. [100]. Moreover, the emergence of real-time ML technologies enables automated anomaly detection during data acquisition, ultimately enhancing the robustness and reliability of ICP-MS as a multi-elemental analytical instrument [101].

A study by Zhang et al. applied ML to investigate the metal sulfide trace elemental properties of metal sulfides in Se–Te-bearing deposits [96]. The study analyzed trace elemental data from chalcopyrite, pyrite, and sphalerite across different kinds of deposit types containing Se–Te. PCA and the silhouette coefficient method approach were used to identify the best optimal end-elements for differentiating genetically distinct deposits, leading to the development of discriminant diagrams. The study revealed the limitations of binary discriminant visualization for ore deposit classification, as indicated revealed by the low accuracy of the SVM in estimating the decision boundaries. The RF model showed that the pyrite RF classification model achieved the best performance, with an accuracy of 94.5% with no overfitting errors. In particular, the feature analysis further showed that selenium (Se) exhibited higher Shapley additive explanations values in epithermal deposits and volcanogenic massive sulfide (VMS) deposits, particularly in the latter, where Se was the most influential important differentiating element factor. In contrast, tellurium (Te) played a greater role in distinguishing Carlin-type deposits, where its contribution, along with Se, was significant. The contributions of Se and Te were comparatively smaller in deposits lower in porphyry and skarn types [96]. 

These results reflect the distinct geochemical behaviors and substitution mechanisms of selenium (Se) and tellurium (Te):

Se: Prefers substituting for sulfur in sulfide lattices (e.g., pyrite, sphalerite) due to similar ionic radii and valence states. It is typically enriched in magmatic–hydrothermal systems such as epithermal deposits and VMS.

Te: Less sulfur-affiliated, commonly forms discrete tellurides (e.g., hessite, calaverite), or concentrates in late-stage hydrothermal fluids. It is often associated with low-sulfidation epithermal and Carlin-type deposits.

pH/Redox: Neutral-to-alkaline fluids enhance Te mobility, while acidic and highly oxidizing conditions favor Se retention.

Temperature: Se concentrations decrease with cooling in VMS systems, whereas Te enrichment peaks during epithermal stages due to fluid boiling.

Exploration targeting: Elevated Te in pyrite halos indicates Carlin-type mineralization potential, while Se-rich zones are indicative of epithermal or VMS prospects.

Xu and Li [97] applied ML algorithms to determine the threshold value for Ba interference with Eu in coal and coal combustion products by ICP-MS. Their study developed ML-based prediction models based on ML for figuring out the threshold value of Ba-Eu interference, considering variables such as Eu, which may be used to forecast this kind of interference in coal. To train the models, Eu, Ba, Ba/Eu ratios, and the degree of interference with Eu were all considered. Experimental validation demonstrated the effectiveness of these various prediction models, and they discovered that the optimal Ba-Eu interference prediction models were: for Ba–Eu < 2950: *y* = − 0.18419411 + 0.00050737 × *X*, 0 < *x* < 2950, when the Ba/Eu value is less than 2950. Furthermore, the best results were obtained using the Ba-Eu interference prediction model of: for 2950 < Ba/Eu < 189, 523: *y* = 0.293982186 + 0.00000181729975 × *X*, 2950 < *x* < 189,523, where the Ba/Eu value is between 2950 and 189,523. A threshold value of Ba/Eu = 363 was identified as suggested based on the optimal below this threshold, Ba interference with Eu can be neglected during Zu data interpretation. Compared with previous studies, the proposed model provides a more accurate estimation of the Ba interference threshold for Eu; that is, when the Ba/Eu value is less than 363 during the interpretation of Eu data, Ba interference with Eu can be disregarded. The suggested prediction model more accurately establishes the threshold value for Ba interference with Eu when this value is compared to those suggested in previous studies [97].

Bishop and Robbins [98] investigated prospective applications of ML for identifying indicators of REE concentrations in sedimentary layers. Using both supervised (regression, SVM, RF, and boosting) and unsupervised (correlation, PCA, and cluster analysis) ML models, the study applied compositional data analysis principles and ML to predict REE abundance in sedimentary lithologies and to explore geochemical relationships. The outcomes from three unsupervised models were consistent, showing that REEs are commonly associated with incompatible elements such as Th, Nb, and Hf. Among the supervised models, with Th and P often being the most significant predictor factors, gradient boosting, Adaboost, and RF achieved the highest predictive performance, with Th and P emerging as the most influential predictor variables, performed the best in forecasting REE concentrations. Understanding novel secondary and unconventional REE sources can be advanced by identifying geochemical indicators of REE enrichment, and this study advances the understanding of secondary and unconventional REE sources, aiding the identification that can be used to help identify potentially exploitable deposits of REE resources based on available data and improving our understanding of metal behavior in sedimentary systems. Although REE concentrations in these sources are typically lower than in primary ore deposits, they remain attractive due to their abundance. The quantity of available feedstock, lower extraction costs, reduced environmental impact, and their contribution to waste remediation and the possibility of easier, less expensive, and less environmentally taxing extraction processes, as well as the extra advantage of cleaning up waste streams and promoting the circular economy, make these sources appealing [98].

A study by Song et al. [99] used three multi-task neural networks to predict REE concentrations in coal ash. The study proposed using ML to estimate the REE contents based on the bulk composition of rare-earth element coal ash, which can be readily measured through standard testing procedures. This provides a multi-task neural network simultaneously predicting the concentrations of the contents of various REEs. Additional model evaluations revealed key data patterns useful for screening coal ash with high REE contents, revealed by additional model evaluations. Furthermore, the study demonstrated that transfer learning can be used to increase the model adaptability's when applied to dealing with coal ash samples from different sources [99].

#### Machine Learning in Laser Ablation–Inductively Coupled Plasma–Mass Spectrometry (LA-ICP-MS)

LA-ICP-MS is an advanced analytical technique used for the precise, spatially resolved quantification of trace elements and isotopes in solid samples [86]. It has numerous wide applications, including in geochemistry, materials science, forensics, and healthcare. Although LA-ICP-MS has great sensitivity and spatial resolution, ML techniques are increasingly being used to analyze and interpret its complex, multidimensional data for effective analysis and interpretation. ML has proven highly effective when integrated, demonstrating significant efficacy when used with spectroscopic methodologies [100]. Models to simulate complex geological data or systems, such as SVM, ANN, DT, and KNN, are commonly used to simulate complex geological data and systems[101]. Despite its success in handling large datasets, ML has been less explored in economic geology. However, it has recently emerged as a transformative approach for mineral characterization through using LA-ICP-MS, enabling the analysis of trace elements in minerals, enabling the classification of distinct mineral kinds and increasing exploration efficiency. Integrating ML algorithms with LA-ICP-MS data has yielded encouraging results in accurately identifying mineral compositions and deposit types. For instance, Sun et al. applied and highlighted the ML (SVM, K-NN, DT, SVM)-based algorithms coupled with mineral geochemistry to classify the Pb–Zn (Qingchengzi) ore deposits in China based on sphalerite data, achieving accuracies for SVM, KNN, and DT of 93.6%, 94.4%, and 98.2%, respectively, demonstrating the effectiveness of ML in distinguishing minerals by predicting the strata-bound and vein-type of mineral ore bodies.

##### Applications of Machine Learning in LA-ICP-MS

(1) Elemental and isotopes classification

SVM, RF, and NN are among the ML classification techniques used to identify samples based on their geochemical fingerprints. For example, in provenance investigations of archaeological artifacts or minerals, ML can distinguish sample groups with similar elemental profiles more effectively than traditional statistical methods [19, 102, 103].

(2) Quantitative modeling

Supervised regression methods, including partial least squares regression, support vector regression, and gradient boosting are increasingly used to enhance the quantitative accuracy of LA-ICP-MS. These models can account for nonlinear effects, instrumental noise, and inter-elemental interference without relying on matrix-matched standards, which are sometimes difficult to obtain [86, 104].

(3) Detecting patterns and anomalies

Unsupervised learning techniques like PCA, t-SNE, and clustering algorithms are used to analyze large datasets to identify patterns, trends, and outliers. These methods are particularly useful for exploratory analysis and for uncovering hidden features in complex sample collections [105].

(4) Automated image and map analysis

Convolutional neural networks (CNNs) and other DL approaches are increasingly being used to automate image picture segmentation, feature detection, and spatial pattern recognition, which aids in linking elemental distributions with structural or compositional pathological characteristics [19].

##### Benefits in LA-ICP-MS Data Interpretation

The interpretation of LA-ICP-MS data is crucial in fields such as geochemistry, environmental science research, material characterization, and forensic analysis. Because the technique enables high-resolution, spatially resolved analysis of trace elements and isotopes in solid samples, it generates large and complex datasets that require robust and reliable interpretation tools [86]. The integration of ML in LA-ICP-MS operations enhances accuracy, automation, and data integration. As computational capabilities improve, the relationship between ML applications and LA-ICP-MS continues to strengthen, with potential approaches including real-time data processing, adaptive sampling, and cloud-based platform integration. Incorporating ML algorithms and multivariate statistical methods has significantly improved LA-ICP-MS workflows and the method's interpretive capabilities. Improvements achieved through RF, support vector regression, and partial least squares regression over conventional univariate calibration methods have led to more precise elemental quantification, making it feasible to quantify elements more precisely [105, 106]. Additionally, these models also provide improved better signal deconvolution and noise reduction, resulting in clearer and higher-quality data signal clarity and data quality. Furthermore, it is also possible to perform automated and high-throughput analysis reducing manual effort, which saves time on interpretation. Robust multivariate pattern recognition is particularly useful, especially for provenance studies, resource classification, and environmental monitoring [100, 107]. Integration with spatial and temporal data is also feasible, allowing for the detection of mineral boundaries, phase distributions, and compositional zonation. This approach further enhances scalability and reproducibility.

#### Machine Learning in XRD Characterization

**Fig. 18:**
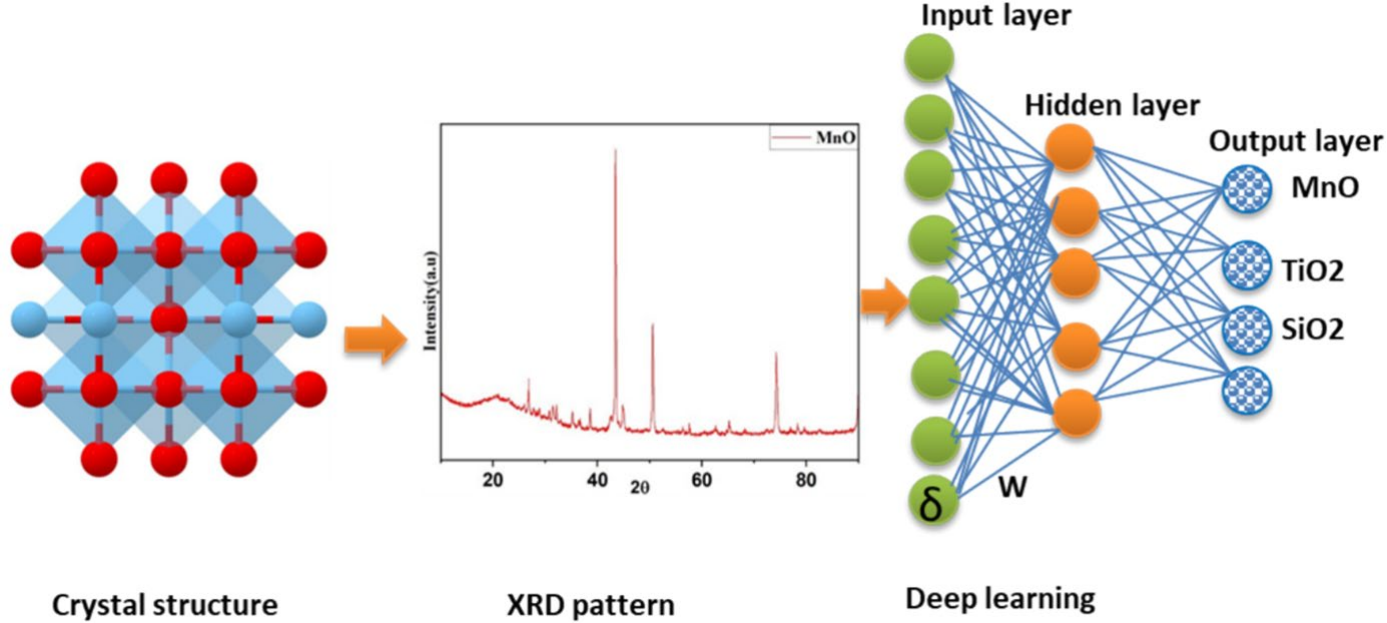
Schematic of AI feed-forward ANN used for analysis of XRD data

XRD is a powerful, non-destructive technique that can offer qualitative and quantitative information about the crystallographic structure and composition of materials. CNN logarithms have the potential to revolutionize XRD analysis by significantly reducing processing times. Demonstrations against using synthetic and real mineral mixture data provide a first assessment of the accuracy of such methods. Neural networks are increasingly applied for XRD analysis of minerals, yielding substantial improvements in phase identification and quantification. These methods use DL approaches to automate processes that have traditionally relied on manual interpretation, both increasing efficiency and accuracy in mineralogical studies. Furthermore, autoencoders and CNNs are increasingly employed for analyzing strain, residual stress, and crystallographic texture in materials through using XRD data [108]. Neural networks can learn directly from unprocessed raw diffraction data, enabling more flexible and precise interpretation compared to conventional methods that depend on predefined models or assumptions [108]. As illustrated in Fig. 18, ANN-based MLPs are used to simplify and classify the XRD data into corresponding metal oxides. The intensity and 2θ data serve are used as inputs, processed through a sigmoid activation function received in the input layer and calculated using sigmoid in the hidden layer to generate the output [109].

The use of CNN and SVM in classifying XRD patterns and crystal structures has shown highly promising results in recent years [110]. CNNs, in particular, have demonstrated superior accuracy over traditional ML models due to their ability to extract spatial features from diffraction data measurements. In some cases, CNN-based approaches have achieved classification accuracies exceeding 95% for crystal structures such as cubic, hexagonal, and orthorhombic [109]. An investigation conducted by Surdu and Győrgy highlighted the growing application of ML techniques that are increasingly applied to XRD for pattern recognition and classification, aiding in phase identification, lattice analysis, and defect detection, thereby advancing the understanding of crystalline materials' microstructural properties [109]. A study by Ukkunda et al. focused on pattern recognition and classification of crystal systems using rendered XRD images, achieving an accuracy of 98–99%. This approach enhanced the performance of ML models compared to traditional tabular XRD datasets [111]. Similarly, Dameli et al. employed CNNs for classifying diffraction patterns in single-particle imaging, using CNNs, focusing on optimizing *F*1-scores and recall, which aided in streamlining the reconstruction pipeline and enhancing pattern classification using an X-ray free-electron laser [112]. In order to quantify phase analysis (QPA) proportions in multiphase materials, ML algorithms such as RF, support vector regression, and NNs have been used to estimate the phase proportions. Research shows that, while these methods enable rapid and automated analysis, their accuracy remains lower than that of conventional techniques, especially when detecting fractions of minor or amorphous phases [113]. Zhang et al. proposed a method approximating coherent scattering power from unit cell parameters and chemical composition, and investigated the QPA using XRD, relying on the proportionality of diffraction intensity to phase abundance. The proposed method approximates coherent scattering power without needing atomic coordinates, allowing for analysis based solely on unit cell parameters and chemical contents. Approximation of the coherent scattering powers per unit cell were determined, with an accuracy within 10% for XRD powder diffraction data without requiring atomic coordinates [114]. Stanko et al. emphasized that QPA using XRD involves determining phase fractions in multiphase samples by analyzing the diffraction patterns. Techniques, including the Rietveld method and doping methods improved, which enhanced the accuracy and reduced systematic errors in phase fraction determination[115]. Jilian et al. correlated α-martensite content with XRD intensities, confirming XRD’S reliability for exploring the QPA, which involved correlating the amount of α′-martensite with XRD intensities through a linear approximation, achieving a coefficient of determination, *R*^2^ of 0.97, indicating a strong correlation despite limited measurement data. In conclusion XRD was able to provide quantitative analysis of phase amounts [116]. ML has also been applied to identify lattice parameters and space groups from XRD patterns. While methods like extremely randomized trees and deep NNs have shown potential promise, the performance depends heavily on varying significantly with dataset quality. For example, crystal systems with fewer training examples (e.g., triclinic) tend to have lower classification accuracy. Renato et al. carried out a study by demonstrating that XRD analysis determines lattice structures by measuring diffraction patterns, which reveal interplanar spacing and atomic arrangements. This technique identifies crystalline phases and defects, supporting applications providing essential data for understanding material properties and facilitating applications in various industries, including microelectronics and mineralogy [117]. A study by Nikita et al. developed a method to model XRD for calculating diffraction effects in crystals with face-centered cubic lattices, focusing on stacking faults and their correlations, utilizing modern methods like differential equations and mathematical modeling to effectively analyze defective structures [118]. Finally, a study by Kano et al. used a CNN with an attention mechanism to extract key XRD peaks, extracting important peaks from XRD spectra to analyze the lattice constants and crystallographic behavior of lithium-ion battery materials, successfully predicting cell voltage by revealing their physical properties and rate performance during charge–discharge cycles using a CNN with attention. The results showed that lattice constants predict cell voltage and reveal rate properties [119].

#### Machine Learning in SEM–EDX

SEM–EDX is increasingly being integrated with ML techniques to improve materials analysis and mineral characterization [120, 121]. The manual evaluation of spectral data required for traditional SEM–EDX analysis relies on spectral evaluation, which can be laborious and subjective. ML models such as SVM, RF, and KNN have been applied to classify minerals, based on the elemental composition profiles derived from EDX spectra [122, 123]. In tasks involving pattern recognition and classification, these algorithms achieved high accuracy in pattern recognition and classification. For example,, RF uses an ensemble of DTs to improve classification robustness, particularly in complex datasets with overlapping features, whereas KNN identifies mineral phases by comparing its spectral features to those of known samples. This integration enables high throughput analysis of complex datasets, making it possible to analyze complicated, heterogeneous geological materials, reducing manual evaluation, which lessens the workload and improves reproducibility [124].

ML models can be trained to detect subtle differences in elemental composition and morphology that are often difficult to identify manually [125, 126]. Large datasets produced during SEM–EDX mapping can also be effectively handled by these algorithms, enabling real-time or nearly real-time mineral identification and quantification [127, 128]. In exploratory mineralogical studies, advanced approaches such as techniques like unsupervised clustering and DL are particularly valuable, since they enable pattern recognition without the need for prior labeling [124]. Furthermore, combining SEM–EDX data with other analytical techniques such as XRD or LIBS provides a more comprehensive analysis, while ML techniques can create a more thorough understanding of the mineral distribution and composition of minerals, which will ultimately help with applications like environmental monitoring, process optimization, and ore characterization [124, 128].

A study by Li et al. [122] applied ML models for mineral classification using SEM–EDS images [123]. In a comparative analysis of mineral classification using SEM–EDS images of a shale sample from the Bakken Formation, five shallow ML models—including logistic regression (LR), linear SVM, KNN, RF, and ANN—were evaluated alongside a DL CNN U-Net model. The models were tuned using grid search, with balanced datasets for the shallow models and original cropped images for U-Net. All models achieved high *F*1 scores (0.86–0.92), with RF performing best among the shallow models (*F*1 = 0.92). Sensitivity analysis revealed LR and SVM to be were more robust to dataset reduction, while RF, NN, and ANN were more affected. RF performance declined most with noise introduced in silicon data, which had the strongest impact on the predictions. Although the U-Net model struggled with minority class segmentation due to class imbalance, it outperformed RF when applied to unseen shale samples, demonstrating its superior generalization capability [122].

Advanced models like CNNs and ANNs further enhance SEM–EDX analysis by learning complex, non-linear relationships between spatial image features and elemental data. CNNs perform particularly well when SEM micrographs are combined with EDX data, enabling the simultaneous analysis of chemical and morphological information. Furthermore, when dealing with high-dimensional spectral data, PCA is frequently applied as a dimensionality reduction step before ML model training. By eliminating noise and focusing on the most useful characteristics, PCA combined with ML algorithms (e.g., PCA + SVM or PCA + ANN) improves both classification speed and accuracy. Overall, ML-enhanced SEM–EDX workflows are becoming essential for rapid, automated, and more accurate mineralogical evaluations across fields such as environmental monitoring, metallurgy, and geosciences. Hao et al. [120] conducted a study using SEM–EDS data and ML algorithms to automatically classify heavy minerals in river sands. The objective of their study was to develop a reliable ML-based classifier capable of identifying various heavy minerals using using EDS data. They used SEM/EDS to acquire the elemental data from 3067 mineral grains representing 22 different heavy mineral types from contemporary river sands. Four classifiers: DT, RF, SVM, and Bayesian network, were compared for classification performance. According to their findings, RF provided the highest classification accuracy, confirming its effectiveness for heavy mineral categorization using SEM–EDX data, as found in ML techniques, particularly RF [120].

Shen et al. [128] conducted a study on DL-based multidefect identification and analysis of SEM images. Their research applied ML techniques to determine the position and morphology of various defect clusters in irradiated steels. They demonstrated that, even with very limited training datasets, a DL-based R-CNN-based DL analysis system can perform comparably to human analysis. This study highlighted the potential of DL for rapid, scalable, and dependable analysis systems for large volumes of contemporary electron microscopy datasets, demonstrating the intriguing potential for using DL to support the development of automated microscopy data interpretation in complex materials, even when numerous features are present [128]. A study by Warmuzek et al. [126] employed a CNN to identify the elements of metal alloy microstructures based on their morphological features. This study demonstrated the effectiveness of a DL approach for recognizing distinct morphological features in alloy microstructures captured through microscopy. The neural network was trained to recognize distinct morphologies, such as spheres, polyhedral, petals, needles, “Chinese script”, twigs, and dendrites, within micrographs of selected cast aluminum and iron alloys. This DL approach effectively distinguished similar structures without relying on fuzzy logic techniques. A key advantage of this method is its adaptability to any set of microscopic images classified into defined categories, eliminating the need for fuzzy logic techniques often required to distinguish visually similar features like twigs and Chinese script. The method’s adaptability to any categorized ser of microscopic images makes it a promising tool for automated analysis and knowledge extraction. Overall, the approach offers a promising and innovative tool for analyzing and extracting knowledge from complex polyphase alloy microstructures [126].

A study by Golsanami et al. [129] applied Lattice–Boltzmann modeling (LBM) and DL to analyze SEM images to characterize clay textures and their influence on the reservoir properties. LBM was applied tor flow simulations with and without clay presence, while SEM images were used for DL-based clay-type identification and quantification. The present study's trained DL model was implemented as a MATLAB application, providing a practical tool that can be used in the future as a handy tool for clay characterization. Using AlexNet transfer learning, the model achieved over 95.4% accuracy on unseen SEM images when applied to unexpected photos after being trained on 2160 images of various clay minerals. The results demonstrated that clays could reduce reservoir permeability and porosity by 400 mD and porosity by more than 30%, respectively. This current study's novel approach provides new insights into the complex methodology, offering fresh perspectives on the intricate effects of clay minerals on the quality of the reservoir. Additionally, the study introduced a depth-slicing method for 2D SEM images, enabling 3D reconstruction and analysis of conventional processing of standard SEM data [129].

A study by Kharin et al. [130] explored the use of synthetic data for the detection of nanoparticles in SEM images using DL. The study showed that semi-synthetic datasets can be used to effectively train a DL neural network for nanoparticle detection. The surface-rendered nanoparticles were assigned textures derived from real SEM images, and transfer learning was applied to train the RetinaNet architecture for large-scale particle distribution analysis. The proposed approach may also be useful for image segmentation tasks. Additionally, the developed software has an easy-to-use graphical user interface requiring no programming expertise, enabling researchers to implement and share algorithms easily across different programming environments. The best algorithms can be readily implemented and shared by researchers using any programming language [130]. Chen et al. [131] developed a DL-based method for mineral characterization using SEM image segmentation, demonstrated using samples from the Duvernay Shale in the Western Canada Sedimentary Basin. Their approach used the U-Net DL architecture analysis, originally designed for biomedical imaging, to interpret geological structures in SEM images of shale samples. By introducing a modified weighting function based on local spatial variability, the model enhanced boundary contrast and effectively differentiated clay aggregates from surrounding mineral matrix particles and organic matter. Trained in TensorFlow for semantic segmentation and feature extraction, the researchers trained the model on 8000 annotated image patches (256 × 256 pixels), derived from larger annotated image tiles. Validation of the model achieved an average intersection-over-union (IOU) of 91.7%, confirming its reliability. When applied to 300 SEM images of Devonian Duvernay Formation shale with varying maturities, the network successfully segmented clay aggregates despite their visual similarity to other minerals. This texture-based DL method offers geoscientists a powerful tool for quantitative mineralogical characterization [131]. Ciresan et al. [132] also automated the SEM image segmentation using a specialized deep ANN as a pixel classifier, demonstrating the feasibility of high-precision, automated mineral feature recognition of deep ANN as a pixel classifier [132]. Table 7 summarizes studies using ML models in SEM–EDX for mineral analysis and characterization.

**Table 7:** Summary of studies using ML models in SEM–EDX for mineral analysis and characterization

Location/study	Type of model used	Outcome	References
Bakken Formation, Williston Basin, North Dakota, USA	Logistic regression, SVM, KNN, RF, ANN, CNN, U-Net	The experimental results showed that RF achieved the highest *F*1 score (0.92); U-Net outperformed RF on unseen samples; noise in silicon had the greater impact on most affected prediction accuracy	[122]
Rock pore structures and permeability studies, North China	DL system	Addressed the drawbacks, overcoming the limitations of conventional image identification methods, such as including low accuracy and poor segmentation, as well as the difficulty of fully capturing the pore space characterization in SEM images	[133]
Alloy microstructure study, Poland	Deep neural networks (custom architecture)	Successfully classified multiple alloy microstructures (e.g., sphere, needle, dendrites); offered an effective alternative to fuzzy logic methods	[126]
Indus Basin, Pakistan	SVM and particle swarm optimization algorithms	The joint inversion approach provided the most reliable prediction of acoustic impedance and porosity distribution in heterogeneous materials. The findings demonstrate that, in the Lower Goru heterogeneous reservoir of the Sawan Gas Field, Pakistan, the joint inversion approach produces the best reliable forecast of AI and porosity distribution	[134]
Duvernay Formation, Canada	U-Net (DL) system	Achieved 91.7% IOU; accurately segmented clay aggregates from SEM images despite similar gray-level intensities to the surrounding matrix particles	[131]

### Machine Learning in Hyperspectral Imaging

**Fig. 19:**
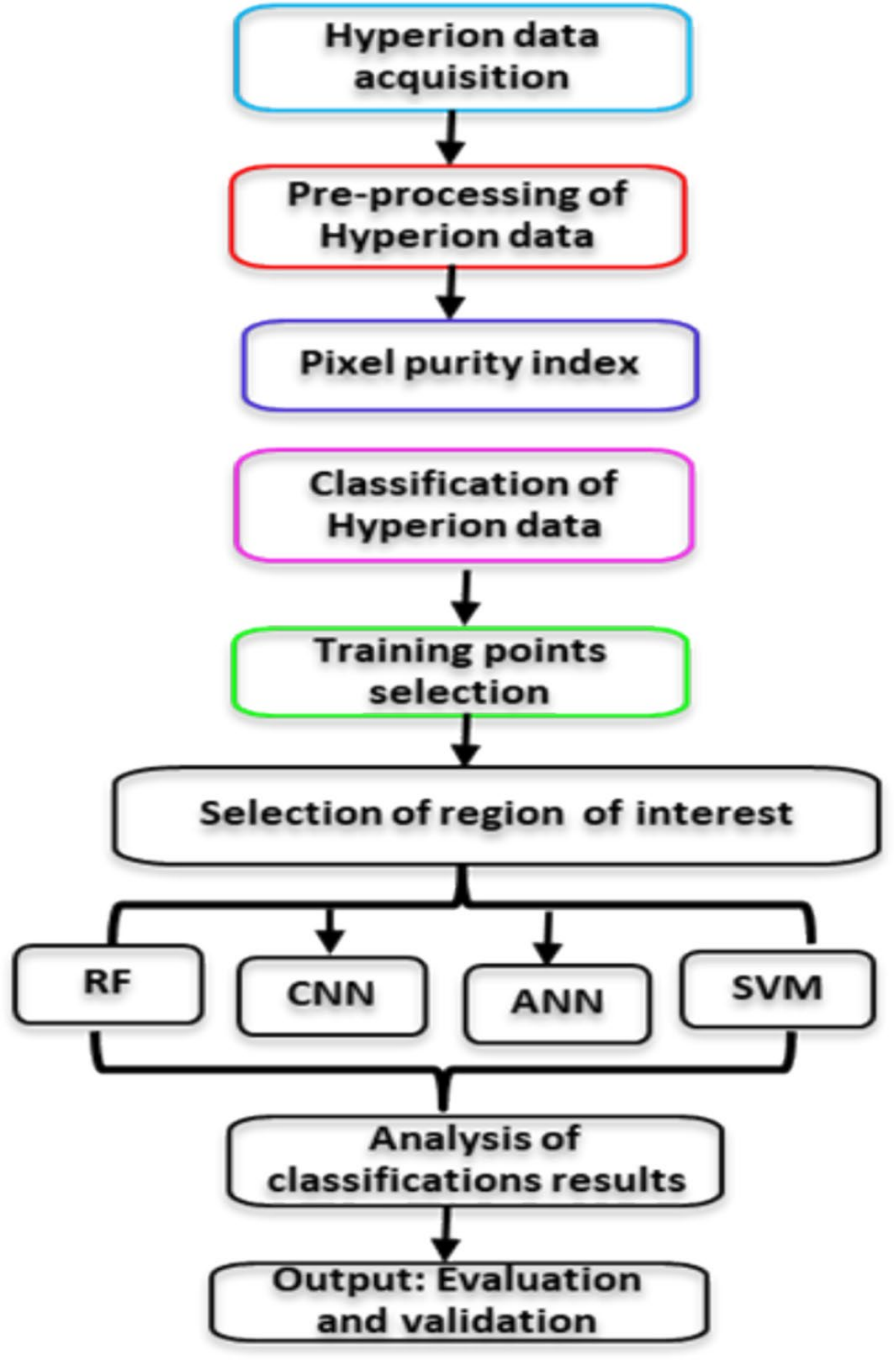
Flow chart summarizing the processing steps used for Hyperion image classification, redrawn from [140].

The incorporation of hyperspectral characterization into mineral analysis characterization and the broader mining industry represents a shift in mineral analysis and how mineralogical and geometallurgical data are applied for processing, exploration, and operational optimization [135]. Its applications include mineral identification, ore sorting, core logging, and geometallurgical modeling. In the mining industry, this technique has emerged as a non-destructive analytical approach for mineral characterization, by capturing detailed spectral data across a wide wavelength range, the identification of which allows the mineralogical and textural properties in geological rocks to be supported by the unique spectral signatures [136]. These signatures, absorbance, reflectance, near infrared, visible, and shortwave infrared spectra, enable precise mineral identification, mineralogical characterization, and lithological analysis composition [137]. Each spectral response provides critical insights into the composition, distribution, and chemical properties of the analyzed materials, presenting challenges that limit conventional analytical methods [138]. For instance, traditional statistical approaches often suffer from the “curse of dimensionality”, leading to overfitting and reduced performance. Additionally, the large data volumes produced by hyperspectral data for certain applications from UAV and airborne platforms make real-time processing difficult [137]. AI tools, specifically ML, SVM, DL CNN, ANN and autoencoder models, facilitate rapid models, allow fast interpretation by spatial–spectral representation and performing effective non-linear transformations, dimensionality reduction, and feature selection to name a few [139]. By integrating modern remote sensing techniques with AI, researchers can address long-standing challenges in lithological identification, especially in geologically complex environments [140]. The use of hyperspectral remote sensing datasets acquired from satellite and airborne platforms has proven to be significant in overcoming common obstacles encountered in mineral exploration and mapping activities [37]. For instance, Yu et al. proposed a 3D convolutional autoencoder for lithological mapping, and the spatial–spectral features were extracted using both pixel-based and cube-based 3D CNN convolutional neural network architectures [139]. Figure 19 summarizes the methodology flowchart for the processing steps used in Hyperion image classification.

### Machine Learning in the Petrographic Microscope

**Fig. 20:**
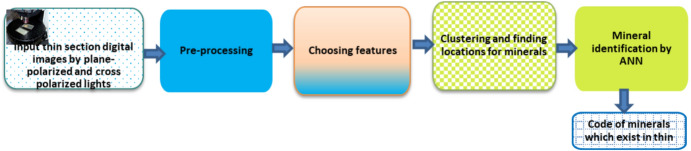
Schematic of **a** a conventional microscope and a modern portable microscope for image acquisition and analysis, and **b** an emerging method based on using a compact, user-operated microscope that transfers analytical data being transferred over the internet for processing by deep ML algorithms based on artificial neural networks [145]

In mineral characterization, petrographic microscopy plays a crucial role by enabling detailed examination of mineral composition, texture, grain size, and mineral intergrowths in geological samples under a polarized light [141]. Petrographic microscopy, also known as polarizing microscopy, has been fundamental in mineral characterization over the past two decades, particularly for applications in image acquisition and analysis. It includes a range of methods, including dark-field, bright-field, phase-contrast, and fluorescence microscopy, which allow the examination of thin rock sections (~ 30 µm thick) where individual mineral grains are separated and their optical properties can be clearly distinguished [142]. In this process, each mineral exhibits distinct interference colors, aiding in its identification and revealing rock textures even if they are often invisible in hand specimens [143]. Petrographic microscopy is widely applied in various applications, including mining, petroleum geology, exploration geology, environmental science, and forensic investigations [144]. With technological advancements and the development and emergence of ML tools, there has been interesting research into IoT-based microscopy [146]. The integration of IoT and ML in microscopy is attracting great attention and is a promising research discipline, allowing automated and efficient analysis of microscopic images, offering a promising direction for future research [145]. Recent innovations, such as compact 3D-printed devices connected via the IoT for data sharing and cloud computing, along with automated image processing using DL algorithms, have the potential to enhance conventional imaging workflow procedures. Despite these advances, significant progress in digital image processing and the interpretation of petrographic data within the optical and geochemical petrological framework of thin sections remains largely manual and has predominantly continued to be an analogue attempt in geochemistry [145] (Fig. 20)

## Comparative Advantages and Disadvantages of AI/ML Methods in Analytical Characterization Techniques

ML/AI algorithms have significantly improved the analytical characterization of minerals; however their efficiency differs, depending on each analytical technique, dataset characteristics and structure, and specific analytical goals [146]. As mentioned, AL/ML algorithms like SVM, CNN, ANN, and RF show distinct advantages and limitations compared with analytical characterization techniques such as ICP-OES, XRF, ICP-MS, and LIBS, to name a few. The common AI logarithms used are SVM, which is effective for spectroscopic characterization like XRF, and LIBS where the datasets are limited in size and typical dimensions. The effectiveness of other AI algorithms are summarized in Table 8, highlighting their strengths and limitations.

**Table 8:** Summary of strengths and limitation Al/ML methods I analytical characterization techniques for minerals

AI/ML logarithm	Analytical characterization technique	Advantages	Limitations	References
SVM	ICP-OES	Efficient for predicting recoveries of target analytes while managing nonlinear spectral patterns, and it handles moderate datasets. Effective in identifying complex features in large datasets, in contrast to conventional methods like partial least squares in mineral characterization when integrated with other spectroscopic datasets. Assists with capturing nonlinear spectral patterns. Used in nonlinearities, interpretable through feature importance	Performance diminishes on large spectral datasets or on interferences that are complex. High data demanding and computationally skill requirements. Less accurate for continuous spectral mapping.	[147] [148]
CNN				
RF				
SVM CNN RF	XRF	Very efficient for high-dimensional spectral datasets, and effective for element description, concentrations, and reduced computational prices. CNN, specifically in XRF, can extract spectral features from imaging XRF data and is effective for automated feature selection. This typical logarithm is robust to overfitting and interpretable.	Not efficient on large datasets and very sensitive to noise and data scaling. Requires a large labeled dataset, has relatively high computational cost, and requires extensive training data with potential shortcoming in auto-identifying less visible lines.	[149] [150]
SVM and CNN RF	ICP-MS	Enhances the accuracy and efficiency in fault prediction, obtaining up to 99% accuracy in material defect detection. Robust against overfitting and capable of handling large datasets with high dimensionality, making it more suitable for numerous classification roles	High skills requirement, computationally intensive, requiring significant resources for optimization and training. Not easily interpretable compared to simple methods, making the process of decision-making more challenging.	
SVM CNN RF	LIBS	This AI algorithm is more effective for rapid elemental classification and suitable for real-time analysis. It has superiority for quantification of multi-element and spectral-image analysis. Stable and easily interpretable and handles noisy spectra.	Performance declines with complex spectral datasets. Requires extensive training of large datasets. Shows less efficiency in capturing spatial–temporal spectral complexity	[151] [152]

## Economic Benefits and Policy Development of AI in the Mining Process and Mineral Characterization

The integration of AI and ML into mining processes and mineral characterization offers substantial economic benefits but also requires careful policy development. These technologies enhance productivity, reduce operational costs, and improve safety while also addressing environmental concerns associated with mining activity [153]. The main economic benefits in mining processes and mineral characterization include: (1) enhanced efficiency, (2) cost reduction, and (3) safety improvement through automation and real-time assessment. Additionally, the predicted net return value (NRV), which is a critical metric for making investment decisions, must be taken into account while evaluating the economic strategy of ML and AI adoption. Specifically, NRV accounts for initial (i.e., first stage) capital costs that are associated with sensors, big data infrastructure, software development, and upskilling the workforce with advanced skills related to AI and ML. All these expenditures must be weighed against the expected financial gains, including the improved throughput, minimal operational risks, improved resource recovery, low energy consumption, and reduced downtime. Measuring the NRV allows the mining sector and metallurgical industries to evaluate the long-term economic viability of AI- and ML-driven solutions and supports strategic investment planning. Policy development should focus on: (1) regulatory frameworks, (2) alignment with sustainability goals, and (3) investment in workforce training for advances of the sophisticated technologies. Research has examined the efforts focused on policy developments and economic viability and the policy implications of implementing AI, ML, and autonomous robotic systems in mining processes. The economic benefits and challenges of AI and ML, and robotics were explored by Hyder et al. [52]. Furthermore, robust policy frameworks are essential to ensure that the development is critical for guiding the ethical application of AI and ML, minimizing potential biases and environmental impacts in mining. Creating regulatory frameworks ensures that these technologies be used appropriately, mitigating any biases and environmental consequences. Engaging in workflow training for employees is equally critical to equip personnel with the required skills needed to collaborate efficiently with advanced technologies, enhancing their benefits. Aligning these policies with sustainability objectives encourages responsible mining practices that mitigate environmental impacts. Jooshaki et al. [154] and co-workers conducted a comprehensive systematic review on applying ML to mineralogical data, highlighting and emphasizing the importance of training of the workforce [148]. These studies have significantly contributed to the advancement of AI and robotic systems in mining operations and mineral identification, while addressing multiple different aspects of operational efficiency and environmental management.

## Conclusion and Future Prospects

The advancement of AI and robotics in mining processes and mineral analytical characterization has been explored. Datasets extracted from Scopus were analyzed, and simulation of these data, using R Studio and VOSviewer, was effectively performed for bibliometric evaluation. The bibliometric analysis indicates that the field is maturing, with growing interdisciplinary collaboration and a positive trend in research focused on, highlighted by keywords such as ML integration with exploration, GIS, remote sensing, and analytical mineral characterization. However, international collaboration remains limited, and the study highlights an opportunity for Africa, particularly South Africa, to strengthen their international global partnerships, enhance skills, and adopt advanced technologies advancements from well-established countries. Robotic–human collaboration has shown notable growth in implementation across several countries. This review reveals that modern mining processes using AI and ML tools—such as artificial neural networks, decision trees, principal component analysis, convolutional neural networks, support vector machines, and *k*-nearest-neighbors—are increasingly revolutionizing mining and mineral characterization. These technologies demonstrate strong potential for optimizing AI and ML in mineral exploration, accurately predicting ore grades, while robotic systems exhibit advanced automation in mineral sampling, thereby enhancing ensuring safety and high productivity, and simplifying complex data-driven decision-making. Novel initiatives and the recent developments of AI applications in analytical chemistry mainly focus on method development, optimization for analytical characterization of complex mineral matrices, improving diagnostic precision, and accelerating the discovery of new characterization techniques. AI is also employed in procedures like chromatography and spectroscopy to better understand complex data and detect patterns that are difficult to identify manually. Nevertheless, full AI integration faces significant challenges, including modeling major obstacles. The interpretability of AI models, data ethics, and cyber-security emerge as significant challenges that must be addressed on an ongoing basis. Furthermore, the requirement for large, high-quality datasets underscores the importance of data integrity and standardization to ensure accurate and scalable outcomes for algorithm training, emphasizing the need of data quality and integrity in achieving accurate findings. Additionally, despite these promising advancements and integrations, several research gaps remain, often due to a lack of standardization, data variability, and limited access to high-quality datasets, bottlenecks that hinder model generalization and scalability. Furthermore, progress in integrating AI and ML in mineral characterization, as well as robotic systems, are constrained by high costs and challenges related to integration with advanced analytical instrumentation. Future research should address these bottlenecks through adaptable ML models, open data initiatives, and more affordable robotic systems. To strengthen research, enhancing technological scalability, promoting commercialization and robust industry–academic partnerships, and investing in workforce development are essential. Ultimately, the integration of AI and ML has the potential to transform mining operations and mineral characterization, paving the way toward safer, more efficient, and sustainable mining practices.

## Data Availability

No datasets were generated or analyzed during the current study.
